# PGC-1α inhibits the NLRP3 inflammasome via preserving mitochondrial viability to protect kidney fibrosis

**DOI:** 10.1038/s41419-021-04480-3

**Published:** 2022-01-10

**Authors:** Bo Young Nam, Jong Hyun Jhee, Jimin Park, Seonghun Kim, Gyuri Kim, Jung Tak Park, Tae-Hyun Yoo, Shin-Wook Kang, Je-Wook Yu, Seung Hyeok Han

**Affiliations:** 1 Department of Internal Medicine, College of Medicine, Institute of Kidney Disease Research, Yonsei University, Seoul, Korea; 2 Severance Biomedical Science Institute, College of Medicine, Yonsei University, Seoul, South Korea; 3 Division of Nephrology, Department of Internal Medicine, Gangnam Severance Hospital, Yonsei University College of Medicine, Seoul, Korea; 4 Oral Cancer Research Institute, Yonsei University College of Dentistry, Seoul, Korea; 5 MET Life Science, Seoul, Korea; 6 Department of Internal Medicine, College of Medicine, Severance Biomedical Science Institute, Brain Korea 21 PLUS, Yonsei University, Seoul, Korea; 7 Department of Microbiology and Immunology, Institute for Immunology and Immunological Diseases, Brain Korea 21 PLUS Project for Medical Science, Yonsei University College of Medicine, Seoul, Korea

**Keywords:** Mechanisms of disease, Experimental models of disease

## Abstract

The NLRP3 inflammasome is activated by mitochondrial damage and contributes to kidney fibrosis. However, it is unknown whether PGC-1α, a key mitochondrial biogenesis regulator, modulates NLRP3 inflammasome in kidney injury. Primary renal tubular epithelial cells (RTECs) were isolated from C57BL/6 mice. The NLRP3 inflammasome, mitochondrial dynamics and morphology, oxidative stress, and cell injury markers were examined in RTECs treated by TGF-β1 with or without *Ppargc1a* plasmid, PGC-1α activator (metformin), and siPGC-1α. In vivo, adenine-fed and unilateral ureteral obstruction (UUO) mice were treated with metformin. In vitro, TGF-β1 treatment to RTECs suppressed the expressions of PGC-1α and mitochondrial dynamic-related genes. The NLRP3 inflammasome was also activated and the expression of fibrotic and cell injury markers was increased. PGC-1α induction with the plasmid and metformin improved mitochondrial dynamics and morphology and attenuated the NLRP3 inflammasome and cell injury. The opposite changes were observed by siPGC-1α. The oxidative stress levels, which are inducers of the NLRP3 inflammasome, were increased and the expression of TNFAIP3, a negative regulator of NLRP3 inflammasome regulated by PGC-1α, was decreased by TGF-β1 and siPGC-1α. However, PGC-1α restoration reversed these alterations. In vivo, adenine-fed and UUO mice models showed suppression of PGC-1α and TNFAIP3 and dysregulated mitochondrial dynamics. Moreover, the activation of oxidative stress and NLRP3 inflammasome, and kidney fibrosis were increased in these mice. However, these changes were significantly reversed by metformin. This study demonstrated that kidney injury was ameliorated by PGC-1α-induced inactivation of the NLRP3 inflammasome via modulation of mitochondrial viability and dynamics.

## Introduction

Chronic kidney disease (CKD) is a global health problem and its prevalence has been increasing worldwide [1–3]. Patients with CKD have an increased risk for progression to end-stage kidney disease and mortality [4, 5]. It is therefore important to find effective therapeutic targets to prevent the progression of CKD. Renal tubulointerstitial inflammation and fibrosis are key pathological hallmarks in the progression of CKD [6–8]. Renal tubular epithelial cells (RTECs) are essential for maintaining fluid and electrolyte homeostasis and account for 90% of the kidney mass. RTECs secure abundant mitochondria and have high levels of peroxisomal proliferator-activated receptor-α (PPARα) and peroxisomal proliferator-γ coactivator-1α (PGC-1α) to fulfill their metabolic and functional energy demands [9]. Growing evidence suggests that mitochondrial dysfunction, characterized by a decline in number and the depolarization, swelling, and disruption of cristae, greatly contributes to kidney fibrosis [10, 11]. Damaged mitochondria do not efficiently produce adenosine triphosphate (ATP) and release excessive reactive oxygen species (ROS) and mitochondrial DNA (mtDNA), which consequently trigger downstream inflammatory responses and lead to cell death and tubular injury.

NOD-like receptor family-pyrin domain-containing 3 (NLRP3) is involved in various host innate immune responses to microbial and nonmicrobial stimuli [12, 13]. Several pathogens and endogenous danger signals released from damaged and dying cells activate the NLRP3 and lead to form a protein complex termed “inflammasome” [14]. The NLRP3 inflammasome then induces auto-process and activation of caspase-1, which results in cleavage of pro-cytokines to mature IL-1β and IL-18. Recently, the NLRP3 inflammasome has been implicated in the pathogenesis of kidney injury and fibrosis [15–17]. In various animal models of kidney disease, the NLRP3 inflammasome pathway is activated and its final products, IL-1β and IL-18, can cause kidney tubule injury [16–19]. Interestingly, renal intrinsic cells such as RTECs and podocytes express NLRP3, suggesting the potential role of NLRP3 inflammasome signaling on cellular injury [20, 21]. Moreover, recent studies have demonstrated the potential interaction between mitochondrial dysfunction and NLRP3 inflammasome activation in renal tubular injury models [22–24]. However, it is unknown whether PGC-1α, a key mitochondrial biogenesis regulator, can regulate the NLRP3 pathway via modulating mitochondrial dynamics.

Thus, we investigated the role of PGC-1α in the regulation of the NLRP3 inflammasome activation in RTECs. In particular, we examined whether altered mitochondrial viability and dynamics induced by activation or suppression of PGC-1α can regulate the NLRP3 inflammasome pathway, and thus affect kidney injury.

## Methods

### Cell cultures, treatment of TGF-β1, PGC-1α activator, and transfection to primary RTECs

RTECs were isolated from C57BL/6 mice. A concise method is described in Supplementary Methods. Subconfluent RTECs were FBS-restricted for 24 h, and then the medium was replaced with 1% FBS DMEM medium for the control group and the same medium with TGF-β1 (5 ng/ml) (R&D Systems, Minneapolis, MN, USA) for the TGF-β1 group. RTECs were harvested for RNA and protein analyses at 48 h after media changes. PGC-1α activator, metformin (1 mM) was treated for both control and TGF-β1 groups. Drug doses were determined based on our prior tests. Furthermore, the cells were also transfected with *Ppargc1a* plasmid (1 μg) (Addgene, Cambridge, MA, USA) and *Ppargc1a* small interfering RNA (siRNA), using Lipofectamine 2000 and Plus reagents (Invitrogen, Carlsbad, CA, USA). Next, 6 h after transfection, media were changed to serum-free media, and the cells were incubated for an additional 48 h. To inhibit *Drp1* expression, we knockdown *Drp1* gene with lentivirus containing *Drp1* targeting short hairpin RNA (shRNA) to infect RTECs (kindly gifted from Dr. Yu J, Yonsei University College of Medicine) [25]. To further explore the relationship between NLRP3 inflammasome and mitochondrial injury, we also obtained RTECs from *Nlrp3* knockout mice (kindly gifted from Dr. Yu J, Yonsei University College of Medicine).

### Animal study and treatment

Male C57BL/6 mice (6 weeks old, initial weight 20 g) were purchased from The Jackson Laboratory (Bar Harbor, ME, USA). The animals were maintained in a temperature-controlled room (22 °C) in a 12 h light/dark cycle. All the animals were randomly assigned. One week after arrival, animals were divided into two groups and fed with either a normal diet (ND; *n* = 10) or 0.2% adenine diet (Ade; *n* = 10) for up to 4 weeks. Both ND and Ade groups were also daily treated with an intraperitoneal injection of metformin (250 mg/kg), one week before the diet start. After 4 weeks of ND or Ade, animals were sacrificed and the kidneys were extracted while anesthetized with Zoletil (10 mg/kg) (Virbac, Carros, France). A concise method for preparation of unilateral ureter obstruction (UUO) mice is described in Supplementary Methods. Kidney samples were then immediately frozen in liquid nitrogen and stored at −80 °C until use.

### Real-time quantitative polymerase chain reaction, western blot analyses

The transcript levels of genes including *Ppargc1a*, NLRP3 inflammasome pathway, mitochondrial dynamics, tumor necrosis factor α-induced protein 3 (TNFAIP3), oxidative stress marker, and profibrotic markers were compared by quantitative polymerase chain reaction (qPCR). The primer sequences used in this study were described in Supplementary Table 1. More detailed methods are described in Supplementary Methods. Protein expression levels of the PGC-1α, NLRP3 inflammasome pathway, TNFAIP3, oxidative stress marker, and profibrotic markers were examined with Western blot analyses. Detailed methods and information on antibodies are separately described in Supplementary Methods.

### Assay of NLRP3 inflammasome assembly

To determine the oligomerization of apoptosis-associated speck-like protein containing a caspase recruitment domain (ASC), a disuccinimidyl suberate (DSS) (Gibco, Thermo Fisher Scientific, Waltham, MA, USA)-mediated cross-linking assay was performed as described previously [26]. In brief, RTECs and cells from kidney tissue samples were pelleted by centrifugation and lysed in 0.5 ml lysis buffer containing 20 mM Hepes-KOH, pH7.5, 150 mM KCl, 1% NP40, 0.1 mM PMSF and protease inhibitor cocktail on ice. The cell lysates were centrifuged at 6000 rpm at 4 °C for 10 min. The pellets were washed twice with PBS and then resuspended in 500 µl PBS. The resuspended pellets were cross-linked with fresh DSS (2 mM) for 30 min, and then pelleted by centrifugation at 6000 rpm for 10 min. The cross-linked pellets were resuspended in 30 µl SDS sample buffer and fractionated on 12% SDS polyacrylamide gel followed by immunoblotting with ASC antibody (Cell Signaling Technology, MA, USA).

### Measurement of oxidative stress levels

Oxidative stress (malondialdehyde [MDA]) levels were measured in RTECs and kidney tissues using an MDA assay kit (Abcam, Cambridge, MA, USA). 10 mg of RTECs were homogenized on ice in 300 μl of MDA lysis buffer (Abcam, Cambridge, MA, USA), then centrifuged (13,000 × *g*, 10 min) to remove insoluble materials. 10 ml of plasma were mixed with 500 μl of 42 mM H_2_SO_4_ and 125 μl of phosphotungstic acid solution at RT for 5 min. After centrifuging (13,000 × *g*, 3 min), the pellet was resuspended on ice with 100 μl of double-distilled H_2_O. Then, 200 μl of solution and 600 μl of 2-thiobarbituric acid solution were incubated at 95 °C for 60 min before cooling to RT in the ice bath for 10 min. The intensity of absorbance at 532 nm was proportional to the MDA level.

### Isolation of mitochondria

RTECs were fractionated into cytosol and mitochondria by using a Mitochondria isolation kit (BioVision, Inc. CA, USA) as per the manufacturer’s protocol. In short, cells were collected and washed with 10 ml ice-cold PBS. Cells were centrifuged at 600 × *g* for 5 min at 4 °C and resuspended in 1.0 ml of 1× Cytosol Extraction Buffer. Homogenization was performed on ice and centrifuged at 1200 × *g* for 10 min at 4 °C to remove nuclei and intact cells. The collected supernatant was centrifuged at 10,000 × *g* for 30 min at 4 °C. The resulting pellets were resuspended in 1.0 ml of 1× Cytosol Extraction Buffer and centrifuged at 10,000 × *g* for 30 min at 4 °C to obtain mitochondria. The obtained mitochondria were lysed in 30 μl of Mitochondrial Lysis Buffer and added to 100 ul of enzyme mixture (included in the isolation kit) with 100 μl absolute ethanol. After centrifugation, the resulting pellet was mtDNA. The cytosolic mtDNA was obtained from the supernatant after precipitation with ethanol. The concentration of mtDNA was determined by qPCR assay.

### Assessment for mitochondrial dynamics and function

For visualization of mitochondrial dynamics, immunofluorescent staining for mitochondria, MitoTracker Deep Red (Gibco, Thermo Fisher Scientific, Waltham, MA, USA) was used. RTECs were fixed in 4% paraformaldehyde and blocked in DPBS containing 5% normal rabbit serum and 0.1% Tween-20. Cells were then stained with anti-mouse NLRP3, MitoTracker, followed by AlexaFluor594-conjugated goat anti-rabbit antibodies (Jackson ImmunoResearch, West Grove, PA), and finally, slides were mounted with DAPI and imaged. We next examined mitochondrial structure by standard transmission electron microscopy. Primary RTECs were fixed with a mixture of 2% paraformaldehyde and 2.5% glutaraldehyde overnight, washed, dehydrated, and embedded in a resin according to standard procedures. Mitochondria were examined under a JEOL 1011 microscope (JEOL, Tokyo, Japan). To assess mitochondrial respiration rate, a Seahorse Bioscience ×24 extracellular flux analyzer was used (Seahorse Bioscience, Billerica, MA, USA). For measurement of mitochondrial ROS production, cells were resuspended in Hank’s Balanced Salt Solution after appropriate treatments, and stained with MitoSOX (Gibco, Thermo Fisher Scientific, Waltham, MA, USA) at 37 °C for 20 min. The fluorescence of the cells was measured by a flow cytometer (FACSVerse, BD Biosciences, San Jose, CA, USA). To assay mitochondrial membrane potential, a tetramethylrhodamine ethyl ester (TMRE) Mitochondrial Membrane Potential Assay Kit (Abcam, Cambridge, MA, USA) was used.

## Results

### The alterations in PGC-1α, mitochondrial dynamics, and NLRP3 inflammasome pathway during kidney injury

**Fig. 1:**
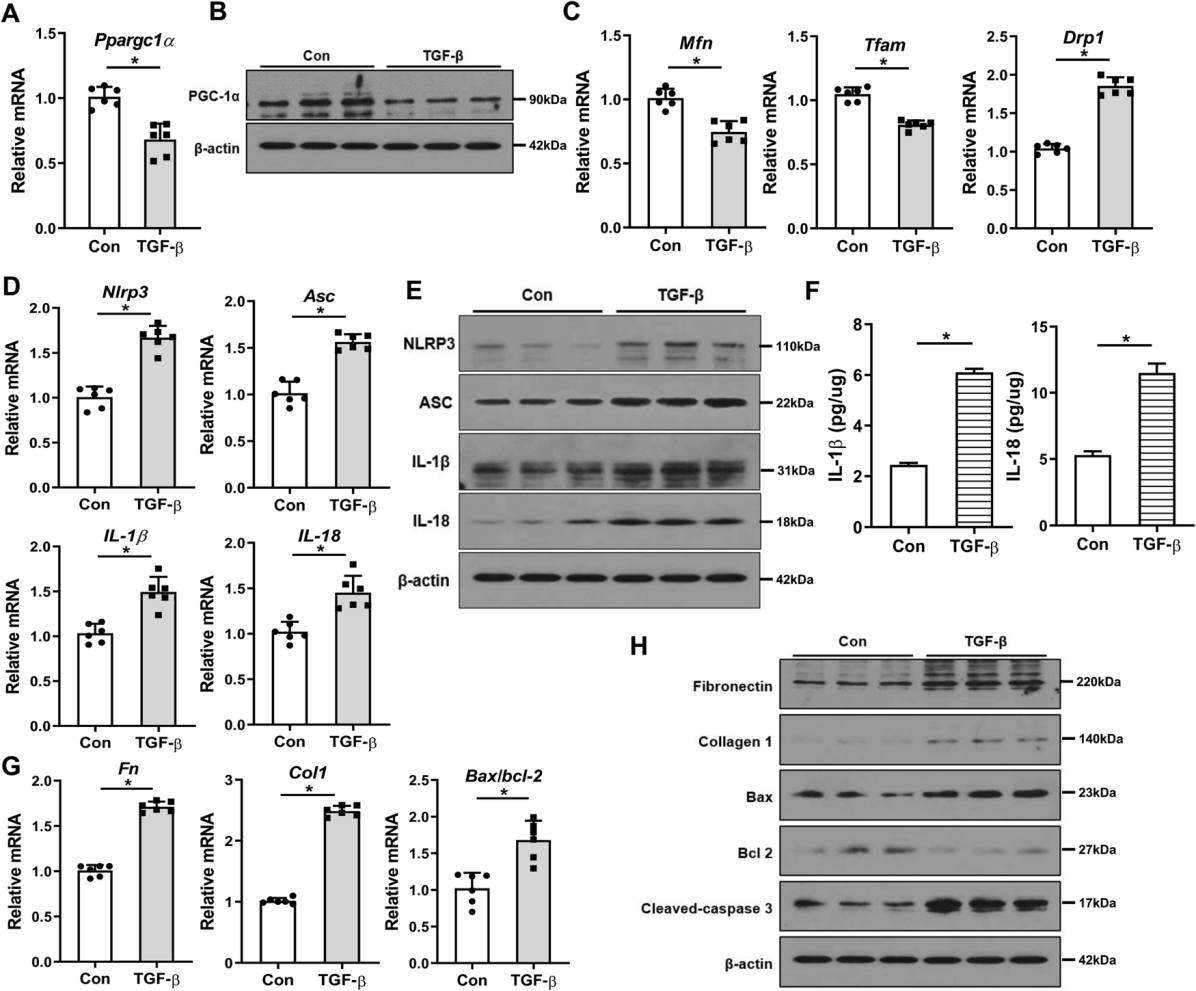
Changes in PGC-1α, mitochondrial dynamics, and NLRP3 inflammasome pathway in TGF-β1-treated RTECs. **A** mRNA and **B** protein expression levels of PGC-1α were decreased in TGF-β1-treated RTECs. **C** mRNA expression of mitochondrial dynamic-related genes including *Mfn*, *Tfam*, and *Drp1*were altered in TGF-β1-treated RTECs. **D** mRNA and **E** protein expression levels of the NLRP3 inflammasome pathway were increased in TGF-β1-treated RTECs. **F** Concentrations of IL-1β and IL-18 assessed by ELISA were increased in TGF-β1-treated RTECs. **G** mRNA and **H** protein expression levels of fibrotic markers including fibronectin and collagen 1, and apoptotic cell death markers of Bax/bcl-2 and cleaved caspase-3 were increased in TGF-β1-treated RTECs. Note: *P < 0.05 vs. control. PGC-1α peroxisomal proliferator-γ coactivator-1α; NLRP3 NOD-like receptor family, pyrin domain-containing 3; ASC, apoptosis-associated speck-like protein containing a caspase recruitment domain; RTEC renal tubular epithelial cell; Mfn mitofusin; Tfam mitochondrial transcriptional factor A; Drp1 dynamin-related protein 1; ELISA enzyme-linked immunosorbent assay.

**Fig. 2:**
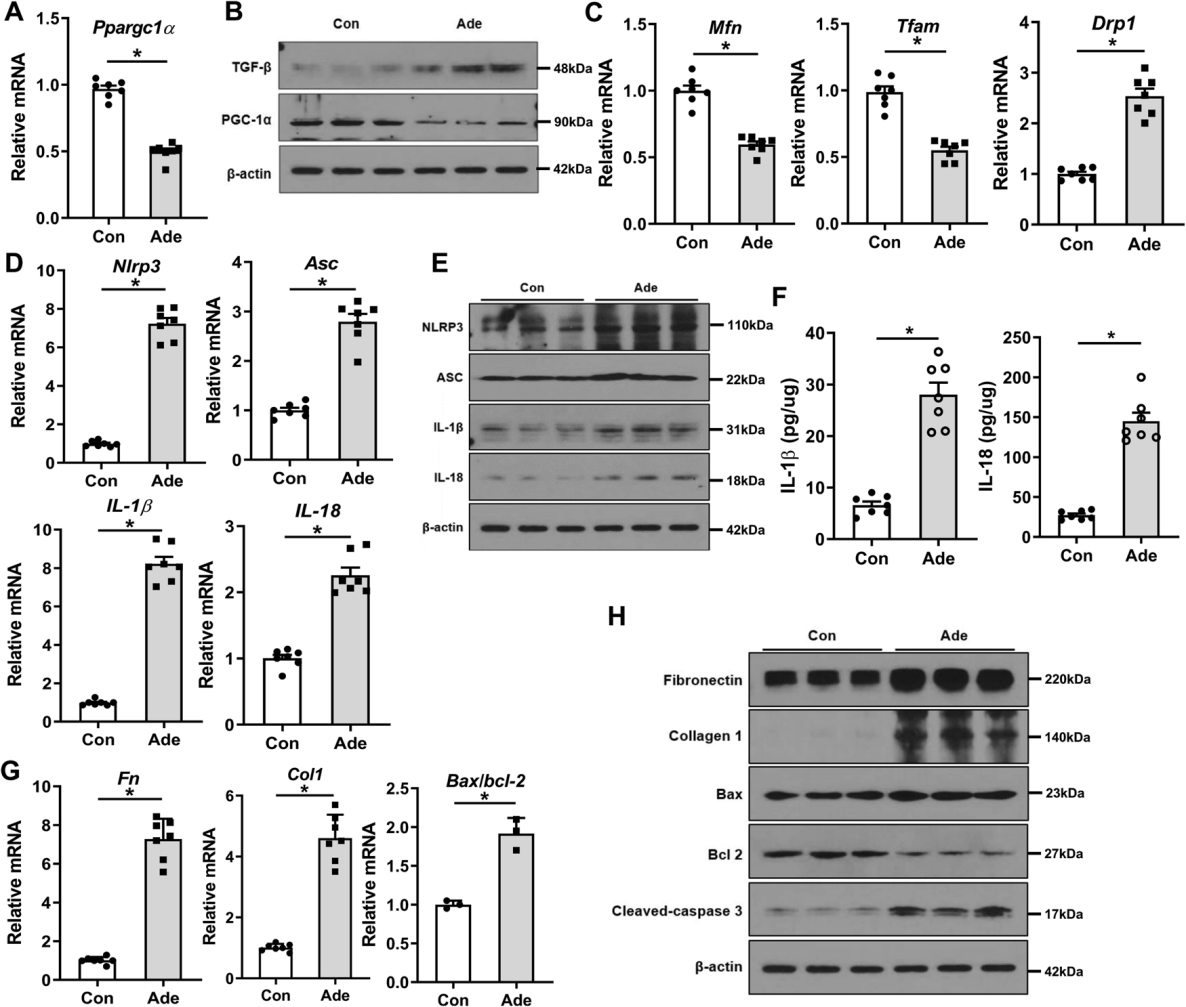
Changes in PGC-1α, mitochondrial dynamics, and NLRP3 inflammasome pathway in adenine-fed mice. **A** mRNA and **B** protein expression levels of PGC-1α were decreased in adenine-fed mice. **C** mRNA expression of mitochondrial dynamic-related genes including *Mfn*, *Tfam*, and *Drp1*were altered in adenine-fed mice. **D** mRNA and **E** protein expression levels of the NLRP3 inflammasome pathway were increased in adenine-fed mice. **F** Concentrations of IL-1β and IL-18 assessed by ELISA were increased in adenine-fed mice. **G** mRNA and **H** protein expression levels of fibrotic markers including fibronectin and collagen 1, and apoptotic cell death markers of Bax/bcl-2 and cleaved caspase-3 were increased in adenine-fed mice. Note: *P < 0.05 vs. control. PGC-1α peroxisomal proliferator-γ coactivator-1α; NLRP3 NOD-like receptor family, pyrin domain-containing 3; ASC, apoptosis-associated speck-like protein containing a caspase recruitment domain; ELISA enzyme-linked immunosorbent assay; Ade adenine.

First, we examined the expression of PGC-1α, mitochondrial dynamics, and NLRP3 pathway in RTECs treated with TGF-β1. In these cells, TGF-β1 treatment decreased the transcript and protein levels of *Ppargc1a* (Fig. 1A, B and Supplementary Fig. 1A). Accordingly, mRNA expression levels of mitofusin (*Mfn*), a mitochondrial fusion-related gene, and mitochondrial transcription factor A (*Tfam*), which represents mitochondrial mass, were significantly decreased, whereas that of dynamin-related protein 1 (*Drp1*), a mitochondrial fission-related gene, was increased compared with control (Fig. 1C). In addition, TGF-β1 treatment activated NLRP3 inflammasome signaling evidenced by increased expression levels of NLRP3 inflammasome pathway-related genes and proteins (Fig. 1D, E and Supplementary Fig. 1B). The concentrations of IL-1β and IL-18, the final products of the NLRP3 pathway, in TGF-β1-treated cell lysates measured by ELISA were increased (Fig. 1F). Furthermore, expression levels of fibrotic markers including fibronectin and collagen 1, and apoptotic cell death index of Bax/bcl-2 ratio and cleaved caspase-3 were also increased (Fig. 1G, H and Supplementary Fig. 1C). These findings were similar in the adenine-induced kidney injury model (Fig. 2A–H and Supplementary Fig. 1D–F). Thus, during kidney injury, there are significant alterations in PGC-1α expression and mitochondrial dynamics, together with activation of the NLRP3 inflammasome pathway.

### PGC-1α restores impaired mitochondrial dynamics and morphology during kidney injury

**Fig. 3:**
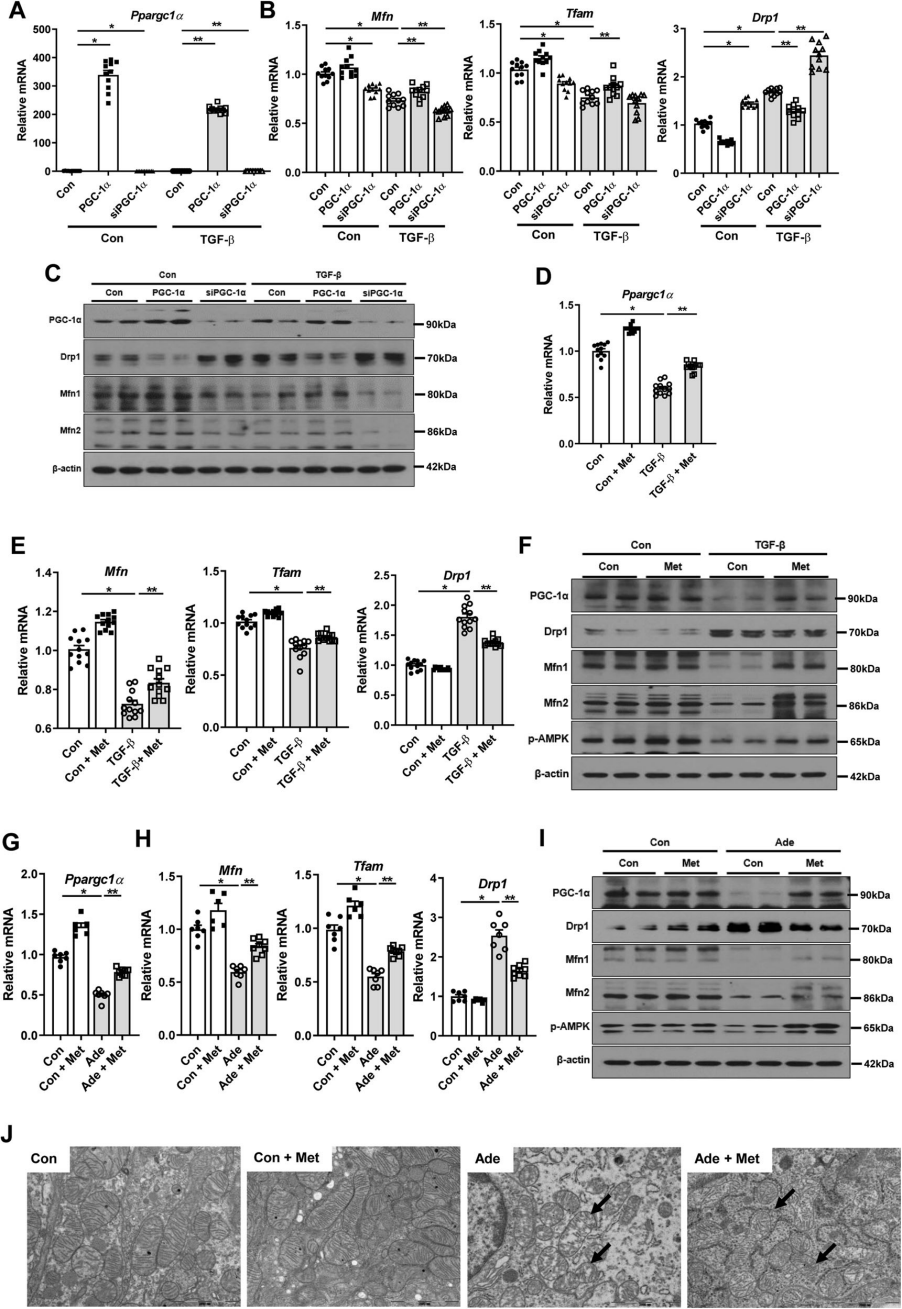
PGC-1α restores impaired mitochondrial dynamics and morphology during kidney injury. **A** mRNA expression level of *Ppargc1a* was modulated by *Ppargc1a* plasmid transfection and siPGC-1α in TGF-β1-treated RTECs. **B** mRNA expression levels of mitochondrial dynamic-related genes were restored by overexpression of *Ppargc1a* with plasmid transfection, whereas reversed by siPGC-1α in TGF-β1-treated RTECs. **C** Protein expression levels of PGC-1α and mitochondrial dynamics were modulated by *Ppargc1a* plasmid transfection and siPGC-1α in TGF-β1-treated RTECs. **D** mRNA expression levels of *Ppargc1a* were increased by metformin in TGF-β1-treated RTECs. **E** mRNA expression levels of mitochondrial dynamic-related genes were restored by metformin in TGF-β1-treated RTECs. **F** Protein expression levels of p-AMPK, PGC-1α, and mitochondrial dynamics were restored by metformin in TGF-β1-treated RTECs. **G** mRNA expression levels of *Ppargc1a* were increased by metformin in adenine-fed mice. **H** mRNA expression levels of mitochondrial dynamic-related genes were restored in adenine-fed mice with metformin. **I** Protein expression levels of p-AMPK, PGC-1α, and mitochondrial dynamics was restored in adenine-fed mice with metformin. **J** Transmission electron microscopy images of RTECs from adenine-fed mice showed restoration of mitochondrial structures with metformin. Note: *P < 0.05 vs. control; **P < 0.05 vs. TGF-β1-treated RTECs or adenine-fed mice. p-AMPK phospho-AMP-activated protein kinase; PGC-1α, peroxisomal proliferator-γ coactivator-1α; RTEC renal tubular epithelial cell; Met metformin; Mfn mitofusin; Tfam mitochondrial transcriptional factor A; Drp1 dynamin-related protein 1; Ade adenine; Met metformin.

Next, we examined whether PGC-1α, a key regulator of mitochondrial biogenesis, could attenuate mitochondrial dynamics during kidney injury. To modulate *Ppargc1α* expression, we additionally used *Ppargc1a* plasmid, siRNA against *Ppargc1α* (siPGC-1α), and metformin, an indirect activator of PGC-1α. As expected, the impaired mitochondrial dynamic-related genes were restored by overexpression of *Ppargc1α*. In contrast, knock-down of *Ppargc1a* with siPGC-1α decreased the expression of *Mfn* and *Tfam*, but increased the expression of *Drp1* (Fig. 3A–C and Supplementary Fig. 2A, B). These findings were corroborated by additional experiments with metformin (Fig. 3D–F and Supplementary Fig. 2C, D). Such improvements led to the recovery of mitochondrial function. Restoration of PGC-1α with *Ppargc1a* plasmid and metformin significantly improved the decreased mitochondrial membrane potential and the decreased oxygen consumption rate in RTECs treated with TGF-β1 (Supplementary Fig. 3A–D). In concordance with changes in PGC-1α, the expression of phospho-AMP-activated protein kinase (p-AMPK) was decreased in TGF-β-treated cells, whereas metformin treatment recovered these expressions (Fig. 3F and Supplementary Fig. 2E). However, metformin did not increase the mRNA and protein expression levels of PGC-1α in RTECs with silencing *Ppargc1a*. These findings suggest that AMPK mediates the effect of metformin and PGC-1α is a downstream effector of AMPK (Supplementary Fig. 2F, G, J). The beneficial effects of metformin on mitochondrial dynamics were also abrogated by silencing *Ppargc1a* (Supplementary Fig. 2H–J). In vivo, treatment with metformin also restored the transcript level of *Ppargc1α* and reversed the altered expression of mitochondrial dynamic-related genes in adenine-fed and UUO mice (Fig. 3G–I and Supplementary Fig. 2K, L, 4A–E). The AMPK activity by metformin was also confirmed in vitro in the adenine-fed model (Fig. 3I and Supplementary Fig. 2M). Electron microscopy examination also confirmed the loss of mitochondria integrity in RTECs of adenine-fed and UUO mice. However, these were significantly improved by metformin (Fig. 3J and Supplementary Fig. 4F). These findings suggest that PGC-1α restores the impaired dynamics, morphology, and functions in mitochondria during kidney injury.

**Fig. 4:**
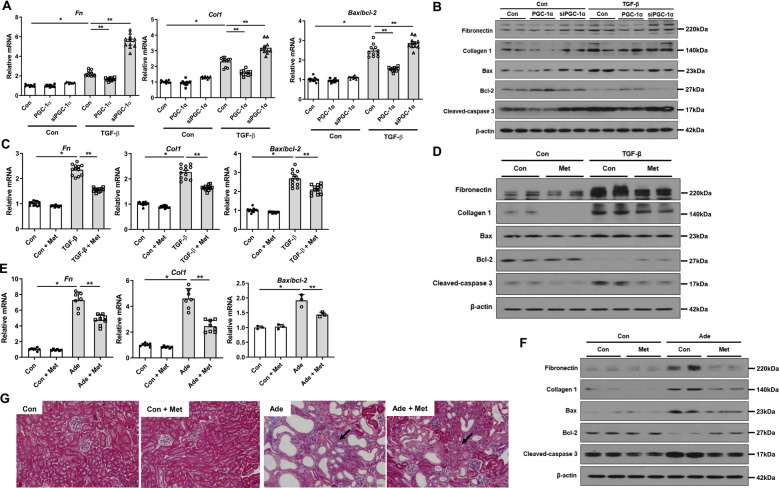
PGC-1α restores cell injury. **A**–**F** mRNA and protein expression of fibrotic and apoptotic markers were attenuated in TGF-β1-treated RTECs with overexpression of *Ppargc1a* and metformin, whereas exacerbated by siPGC-1α. **G** Degree of kidney fibrosis by Masson’s trichrome staining was attenuated in adenine-fed mice with metformin. Note: *P < 0.05 vs. control; **P < 0.05 vs. TGF-β1-treated RTECs or adenine-fed mice. PGC-1α peroxisomal proliferator-γ coactivator-1α; RTEC renal tubular epithelial cell; Ade adenine; Met metformin.

The mitochondrial damage can lead to kidney cell death and fibrosis. We observed elevated expression levels of fibrotic markers including fibronectin and collagen 1, and apoptotic cell death index of Bax/bcl-2 ratio and cleaved caspase-3 in RTECs after TGF-β1 treatment. These increased expressions of cell death markers were attenuated by overexpression of PGC-1α and metformin, whereas exacerbated by siPGC-1α (Fig. 4A–D and Supplementary Fig. 5A, B). In accordance with these findings, the expression levels of fibrotic markers and apoptotic cell death index were significantly increased in adenine-fed and UUO mice. In contrast, metformin reversed all these expressions (Fig. 4E, F and Supplementary Fig. 5C, 6A, B). Masson’s trichrome staining also confirmed the improved fibrosis by metformin (Fig. 4G). Notably, the augmented cell death markers and NLRP3 signaling in RTECs treated with TGF-β were reversed by Z-Asp-2,6-dichlorobenzoyloxymethylketone (Enzo Life Science, Farmingdale, NY, USA), a pan-caspase inhibitor, suggesting that TGF-β-induced injury is likely to be mediated by mitochondrial damage and early apoptosis (Supplementary Fig. 7A–D). These findings together suggest that activation of PGC-1α attenuates mitochondrial damage and decreases cell death and kidney fibrosis.

### PGC-1α modulates NLRP3 inflammasome signaling pathway

**Fig. 5:**
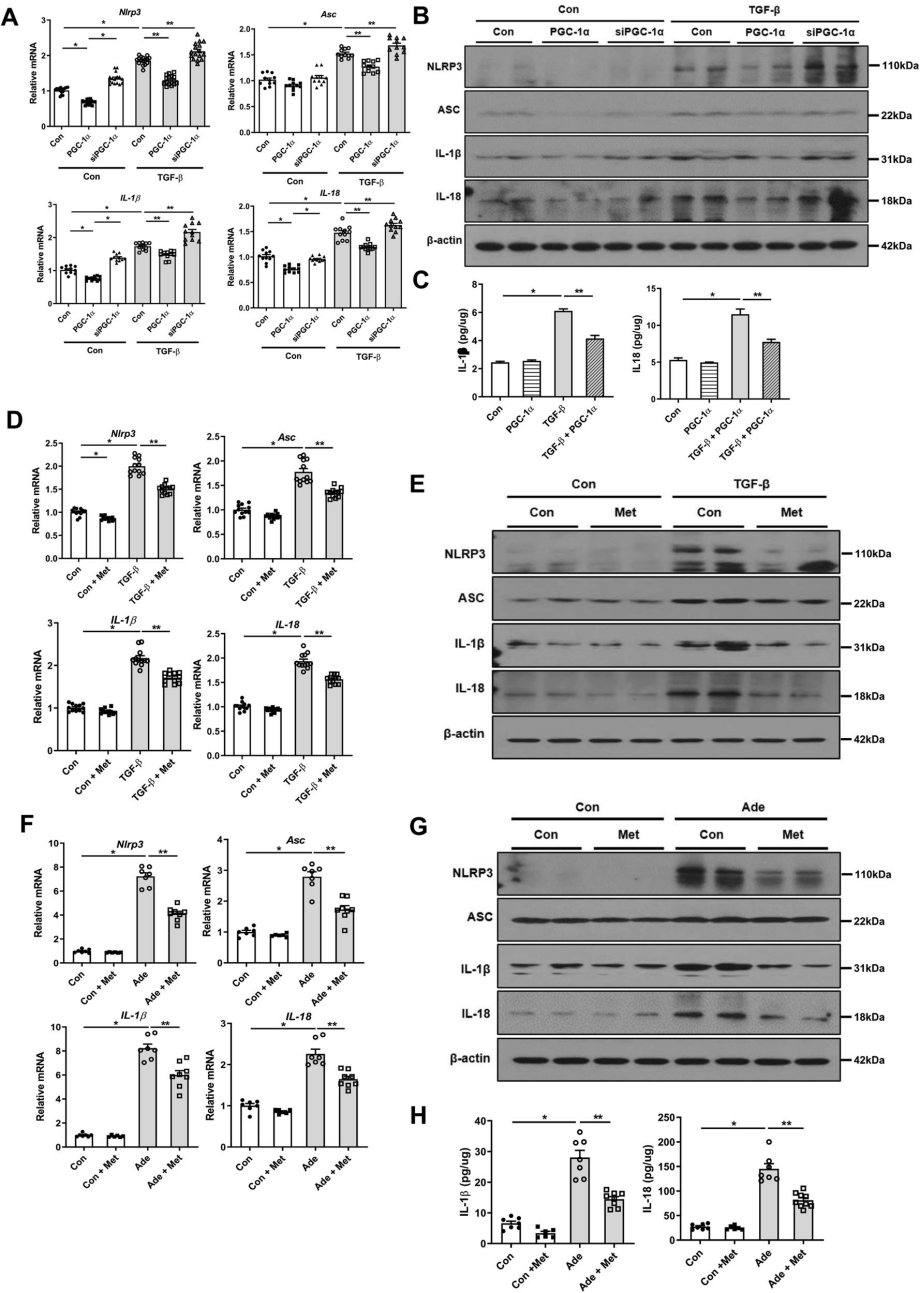
PGC-1α modulates the NLRP3 inflammasome signaling pathway. **A** mRNA and **B** protein expression levels of NLRP3 inflammasome pathway were reduced in TGF-β1-treated RTECs with transfection of *Ppargc1a* plasmid, whereas increased by siPGC-1α. **C** Concentrations of IL-1β and IL-18 assessed by ELISA were reduced in TGF-β1-treated RTECs with overexpression of *Ppargc1a*. **D** mRNA and **E** protein expression levels of the NLRP3 inflammasome pathway were attenuated in TGF-β1-treated RTECs with metformin. **F** mRNA and **G** protein expression levels of the NLRP3 inflammasome pathway and **H** concentrations of IL-1β and IL-18 assessed by ELISA were attenuated in adenine-fed mice with metformin. Note: *P < 0.05 vs. control; **P < 0.05 vs. TGF-β1-treated RTECs or adenine-fed mice. PGC-1α peroxisomal proliferator-γ coactivator-1α; NLRP3 NOD-like receptor family, pyrin domain-containing 3; ASC apoptosis-associated speck-like protein containing a caspase recruitment domain; RTEC renal tubular epithelial cell; ELISA enzyme-linked immunosorbent assay; Ade adenine; Met metformin.

We then further explored the relationship between PGC-1α and NLRP3 inflammasome pathway in mediating kidney injury. When overexpression of PGC-1α was induced using *Ppargc1a* plasmid transfection, the expression levels of NLRP3, ASC, IL-1β, and IL-18 were diminished in TGF-β1-treated RTECs (Fig. 5A, B and Supplementary Fig. 8A). The concentrations of IL-1β and IL-18 in TGF-β1-treated cell lysates measured by ELISA were also decreased after *Ppargc1a* plasmid transfection (Fig. 5C). Treatment with metformin yielded a similar finding (Fig. 5D, E and Supplementary Fig. 8B). However, knock-down of *Ppargc1a* resulted in elevated expression levels of NLRP3 inflammasome pathway-related genes and proteins (Fig. 5A, B and Supplementary Fig. 8A). Moreover, the activation of the NLRP3 inflammasome signaling pathway in adenine-fed and UUO mice was concomitantly decreased by metformin (Fig. 5F, G and Supplementary Fig. 6C, D, 8C). The concentrations of IL-1β and IL-18 in RTECs and the kidney tissue lysates were significantly decreased by metformin treatment measured by ELISA (Fig. 5H).

Given the ability of PGC-1α to regulate mitochondrial dynamics, we then tested the role of Drp1 in NLRP3 inflammasome activation. The NLRP3 inflammasome signaling was significantly reduced by the knockdown of *Drp1*. However, in RTECs with both knockdown of *Ppargc1a* and *Drp1*, the expression levels of NLRP3 inflammasome components were not decreased compared with those in TGF-β1-treated cells. In RTECs with *Ppargc1a* overexpression and *Drp1* knock-down, NLRP3 inflammasome signaling was less activated (Supplementary Fig. 9A, B). These findings suggest that Drp1 can partly mediate NLRP3 inflammasome activation, but other actions of PGC-1α are involved in the regulation of the NLRP3 signaling pathway.

We further examined whether a feedback signal from the NLRP3 inflammasome exists. To this end, we obtained RTECs by primary culture from *Nlrp3* knockout mice. In these cells treated with TGF-β1, there was less activation of NLRP3 signaling than in counterpart cells, which led to the improvements in mitochondrial dynamics (Supplementary Fig. 10A–E). Accordingly, there were concomitant improvements in fibrotic changes and cell death in the absence of *Nlrp3* (Supplementary Fig. 10F, G). These findings suggest a possible bidirectional relationship between mitochondrial damage and NLRP3 signaling.

**Fig. 6:**
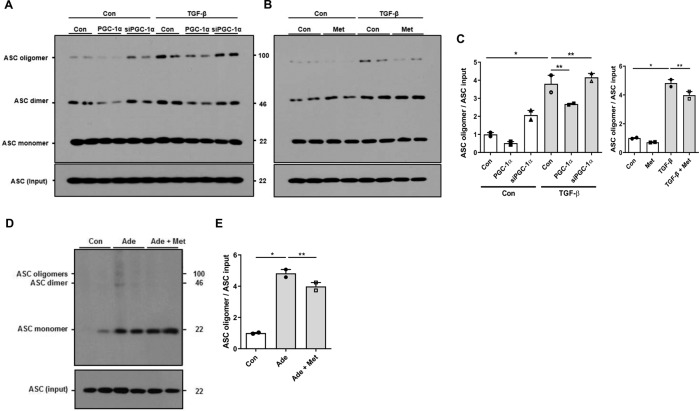
PGC-1α modulates oligomerization of NLRP3 inflammasome during kidney injury. **A**–**C** ASC oligomeric structures assayed by DSS-mediated cross-linking were observed in TGF-β1-treated RTECs, which were attenuated by overexpression of *Ppargc1a* and metformin, whereas aggravated by siPGC-1α. **D**, **E** ASC oligomeric structures were observed in adenine-fed mice, which were attenuated by metformin. Note: *P < 0.05 vs. control; **P < 0.05 vs. TGF-β1-treated RTECs or adenine-fed mice. PGC-1α peroxisomal proliferator-γ coactivator-1α; NLRP3 NOD-like receptor family, pyrin domain-containing 3; ASC apoptosis-associated speck-like protein containing a caspase recruitment domain; DSS disuccinimidyl suberate; RTEC renal tubular epithelial cell; Ade adenine; Met metformin.

Oligomerization of NLRP3 with the adapter protein, ASC, is a key step of inflammasome complex formation. Thus, we examined whether the assembly of NLRP3 inflammasome is affected by PGC-1α. In TGF-β1-treated RTECs, ASC binding to NLRP3 was observed. This oligomerization was significantly decreased by overexpression of PGC-1α or metformin, whereas the binding was restored by siPGC-1α (Fig. 6A–C). In adenine-fed mice, NLRP3 oligomerization and activation were confirmed by ASC binding to NLRP3 and this was abolished by metformin (Fig. 6D, E).

**Fig. 7:**
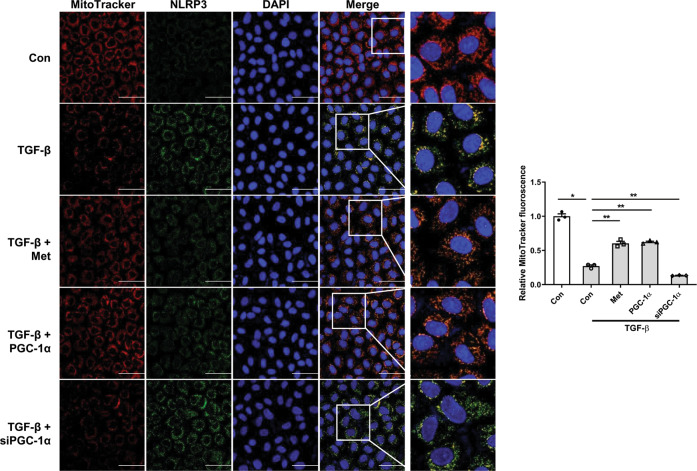
Localization of NLRP3 with mitochondria with or without PGC-1α. Confocal microscopy examination revealed that there was a reciprocal change in MitoTracker Red staining intensity (red) and NLRP3 expression (green) in RTECs with or without PGC-1α However, these findings were reversed by siPGC-1α. The blue signal represents nuclear fluorescence. Note: *P < 0.05 vs. control; **P < 0.05 vs. TGF-β1-treated RTECs. NLRP3 NOD-like receptor family, pyrin domain-containing 3; RTEC renal tubular epithelial cell; PGC-1α peroxisomal proliferator-γ coactivator-1α.

Finally, we further examined mitochondrial contents and concomitant change in NLRP3 expression. Confocal microscopy examination revealed that there was a reciprocal change in MitoTracker Red intensity and NLRP3 expression in RTECs with or without PGC-1α. TGF-β1 decreased the staining intensity of MitoTracker and these were restored by PGC-1α overexpression and metformin. Conversely, the increased expression of NLRP3 in TGF-β1-treated RTECs was reduced by PGC-1α overexpression and metformin. However, siPGC-1α reversed these findings (Fig. 7). In aggregate, these findings indicate that the assembly of the NLRP3 inflammasome complex was induced during kidney injury, and the activation of this pathway was attenuated by PGC-1α.

### PGC-1α orchestrates the release of mtDNA, oxidative stress, and TNFAIP3 to regulate NLRP3 inflammasome

**Fig. 8:**
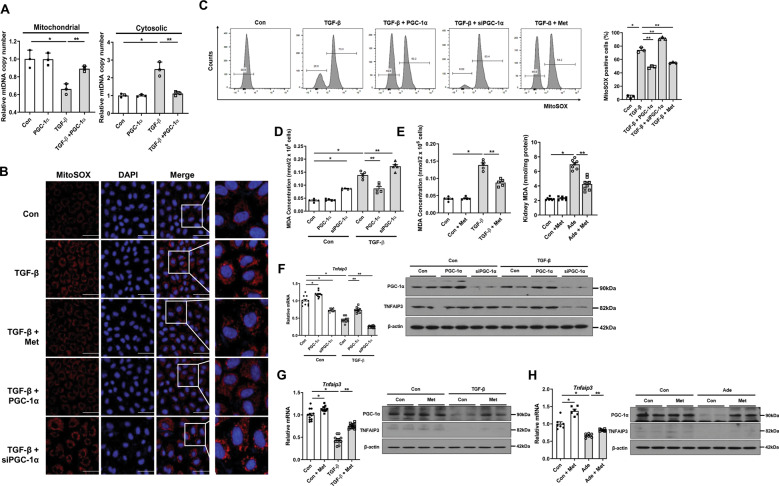
PGC-1α regulates the release of mtDNA, oxidative stress, and TNFAIP3 to regulate the NLRP3 inflammasome. **A** mtDNA copy numbers were decreased in the mitochondrial fraction, while increased in cytosolic fraction in TGF-β1-treated RTECs, which were reversed by *Ppargc1a* overexpression. **B** Confocal microscopy analysis with MitoSOX staining revealed increased production of mitochondria-generated ROS in TGF-β1-treated RTECs, which were reduced with overexpression of *Ppargc1a* and metformin. Conversely, siPGC-1α exacerbated mitochondrial ROS production. **C** Detection of mitochondria-generated ROS in TGF-β1-treated RTECs by FACS analysis. **D** Measurement of oxidative stress levels by MDA showed a reduction of oxidative stress levels in TGF-β1-treated RTECs with overexpression of *Ppargc1a* and metformin, which were increased by siPGC-1α. **E** The oxidative stress levels measured by MDA were reduced in adenine-fed mice with metformin. **F**, **G** mRNA and protein expression levels of TNFAIP3 in TGF-β1-treated RTECs were increased with overexpression of *Ppargc1a* and metformin, which were reduced by siPGC-1α. **H** mRNA and protein expression level of TNFAIP3 in adenine-fed mice was increased with metformin. Note: *P < 0.05 vs. control; **P < 0.05 vs. TGF-β-treated RTECs or adenine-fed mice. PGC-1α peroxisomal proliferator-γ coactivator-1α; mtDNA mitochondrial DNA; NLRP3 NOD-like receptor family, pyrin domain-containing 3; RTEC; renal tubular epithelial cell; ROS reactive oxygen species; MDA malondialdehyde; TNFAIP3 tumor necrosis factor α induced protein 3; FACS Fluorescence-activated cell sorting; Ade adenine; Met metformin.

To further clarify the mechanistic link between PGC-1α and NLRP3 inflammasome pathway in kidney injury, we first examined the changes in mtDNA upon cell injury, which is known as a trigger of NLRP3 inflammasome activation. The mtDNA copy numbers were decreased in the mitochondrial fraction and increased in the cytosolic fraction after TGF-β1 treatment, suggesting that mtDNA was released from the mitochondria into the cytosol. Notably, these changes were restored after overexpression of *Ppargc1a* (Fig. 8A).

Then, we examined oxidative stress, a positive regulator of NLRP3 inflammasome. In TGF-β1-treated RTECs, oxidative stress was notably pronounced compared with controls evidenced by increased MitoSOX staining intensity and increased MDA levels. Treatment with *Ppargc1a* plasmid and metformin attenuated this overproduction of mitochondrial ROS. Conversely, *Ppargc1a* knock-down further increased oxidative stress levels in TGF-β1-treated RTECs (Fig. 8B–D). Similar to the findings of in vitro study, MDA levels were significantly increased in adenine-fed and UUO mice. The enhanced oxidative stress was reduced by metformin (Fig. 8E and Supplementary Fig. 6E).

Lastly, we further examined TNFAIP3, which is regulated by PGC-1α and is also known as a negative regulator of NLRP3 inflammasome. TGF-β1 reduced transcript levels of *Tnfaip3* and this decreased expression of *Tnfaip3* was restored by restoration of PGC-1α. In contrast, *Ppargc1a* knock-down resulted in further decreased expression of *Tnfaip3* (Fig. 8F, G and Supplementary Fig. 8D, E). Moreover, there was a decreased expression of TNFAIP3 in adenine-fed and UUO mice and metformin restored this expression (Fig. 8H and Supplementary Fig. 6F, 8F). In aggregate, these findings suggest that PGC-1α can regulate NLRP3 inflammasome via modulation of mtDNA release, mitochondrial ROS/oxidative stress, and TNFAIP3.

## Discussion

The present study showed that PGC-1α mitigated mitochondrial damage and oxidative stress levels, restored mitochondrial integrity and TNFAIP3, and attenuated the activation of the NLRP3 inflammasome pathway in the TGF-β-treated RTECs and animal models of kidney injury. These improvements concomitantly resulted in decreased cell injury and fibrosis. A schematic figure showing the potential mechanism on the regulation of NLRP3 inflammasome by PGC-1α is presented in Supplementary Fig. 11. The findings of this study unravel the role of PGC-1α in the regulation of the NLRP3 inflammasome signaling via modulating mitochondrial viability and dynamics and also suggest a possible therapeutic potential of PGC-1α for kidney injury.

The NLRP3 inflammasome has been implicated in the pathogenesis of cellular injury, inflammation, and fibrosis in various kidney injury models [15–19, 27–29]. In agreement with previous studies, we showed that the expression levels of NLRP3 inflammasome pathways including NLRP3, ASC, IL-1β, and IL-18 were increased along with elevated expression levels of cellular injury markers in TGF-β1-treated RTECs and animal models with adenine diet and UUO. Several damage-associated molecular patterns (DAMPs) released during renal tubular cell injury are suggested to activate the NLRP3 inflammasome [27, 30–37]. Notably, kidney intrinsic cells express components of the NLRP3 inflammasome pathway [38], and activation of this signaling can contribute to kidney injury [15, 29]. However, it is uncertain how NLRP3 is activated in these cells. In this study, we particularly focused on regulators of mitochondrial biogenesis because dysregulated mitochondria can trigger the activation of the NLRP3 inflammasome pathway [39, 40].

Given abundant mitochondrial contents found in the kidney, growing attention has been paid to PGC-1α in the kidney disease research field. In the kidney, PGC-1α expression is localized to the cortex and outer medulla, corresponding to regions of high mitochondrial activity [41]. As damaged mitochondria are apparently observed in various forms of kidney injury, several studies have demonstrated the crucial protective role of PGC-1α against kidney disease models [41–43]. Furthermore, there has been accumulating evidence that loss of PGC-1α contributes to the development of renal fibrosis and subsequent CKD [9, 44, 45]. In the Notch-induced kidney injury model, PGC-1α also protected tubule injury and ameliorated fibrosis [9]. Here, we demonstrated the role of PGC-1α in preventing kidney injury in light of the regulation of the NLRP3 inflammasome pathway. The overexpression of PGC-1α with plasmid and metformin attenuated TGF-β1-induced cell damage as well as activation of the NLRP3 inflammasome. These results were consistent with adenine-fed and UUO mice models. Conversely, down-regulation of PGC-1α augmented the activation of NLRP3 inflammasome and cellular injury.

To date, few studies have examined the relationship between PGC-1α and NLRP3. In a study by Diao et al. [46], severe burn injury-induced endoplasmic reticulum stress in hepatocytes activated NLRP3 inflammasome. Interestingly, activation of hepatic NLRP3 inflammasome was in parallel with inhibition of PGC-1α. They further examined the upstream regulators of PGC-1α such as protein kinase A catalyst, AMPK, and sirtuin-1, all of which were significantly decreased after burn injury. They suggested that the lack of PGC-1α may play an important role in the metabolic derangement and contributes to the activation of the NLRP3 inflammasome pathway. However, this study did not clarify how PGC-1α is involved in the NLRP3 pathway.

In this study, we demonstrated several mechanisms that PGC-1α regulates the NLRP3 inflammasome pathway in the kidney. Given that PGC-1α plays a key role in regulating mitochondrial biogenesis and mitochondrial dynamics and dysregulated mitochondria trigger the NLRP3 pathway, it can be presumed that PGC-1α could regulate the NLRP3 inflammasome pathway via modulating mitochondrial viability. As shown in our experiments, the expression levels of mitochondrial dynamic-related genes were dysregulated in TGF-β1-treated RTECs. These changes were restored by overexpression of PGC-1α and metformin, while down-regulation of PGC-1α aggravated this dysregulation. The structural improvement of TGF-β1-induced mitochondria damage by PGC-1α was also observed by MitoTracker staining and electron microscopy. The functional assay of mitochondria showed that restoration of PGC-1α significantly improved the reduced mitochondrial membrane potential and the decreased oxygen consumption rate in TGF-β1-treated RTECs. Finally, we showed the release of mtDNA to the cytosol from the mitochondrial fraction in TGF-β1-treated RTECs. This release of mtDNA was prevented by *Ppargc1a* overexpression. It should be noted that mitochondria-driven mtDNA is known to activate NLRP3 inflammasome [39, 47]. These findings together suggest that dysregulated mitochondrial dynamics contribute to the activation of NLRP3 inflammasome and consequent renal tubulointerstitial inflammation and fibrosis. However, there may be a reciprocal interaction between mitochondrial injury and NLRP3 inflammasome. A previous study by Yu et al. demonstrated that NLRP3 inflammasome activation caused mitochondrial damage via multiple pathways [39]. Similarly, we also showed that silencing NLRP3 attenuated the activation of the NLRP3 signaling pathway and led to improvements in mitochondrial dynamics. These findings indicate the possibility of bidirectional interaction between NLRP3 and mitochondria.

PGC-1α also has critical roles in essential metabolic processes such as fatty acid oxidation, oxidative phosphorylation, and ROS detoxification [48–50]. In addition, several NLRP3-activating stimuli are associated with ROS production [32]. Thus, regulation of oxidative stress levels by PGC-1α can affect NLRP3 inflammasome activation. Recently, mitochondrial ROS production has been described to trigger activation of NLRP3 inflammasome during renal tubulointerstitial fibrosis. Zhuang et al. [22] reported that mitochondrial-derived oxidative stress mediated albumin-induced mitochondrial dysfunction and subsequent renal tubular injury. Furthermore, NLRP3 inflammasome was activated in the kidney by albumin overload, which was entirely abolished by MnTBAP, a mitochondrial ROS scavenger. In line with these findings, we showed that enhanced oxidative stress was associated with the activation of the NLRP3 inflammasome pathway during kidney injury. This increased oxidative stress level was attenuated by overexpression of *Ppargc1a* and metformin, whereas augmented after down-regulation of *Ppargc1a*. The reduction of oxidative stress levels by metformin was also confirmed in two different animal models. Taken together, enhanced oxidative stress levels including mitochondrial ROS production by PGC-1α deficiency can result in the activation of NLRP3 inflammasome and consequent renal tubulointerstitial inflammation and fibrosis.

TNFAIP3 is known to be directly regulated by PGC-1α and it has a role in inactivating the NLRP3 inflammasome pathway in inflammatory cells such as macrophage. Kang et al. [51] showed that dysfunctional telomeres cause macrophage mitochondrial distress, metabolic imbalance, and hyperactivation of the NLRP3 inflammasome. They identified the PGC-1α/TNFAIP3 axis as a mechanism responsible for the homeostatic role of the telomere, and the disturbance in this axis led to inflammatory *Terc*^−^^/−^ macrophages and severe bacterial pneumonia in *Terc*^−^^/−^ mice. In the present study, TGF-β1 decreased the transcript level of *Tnfaip3* in RTECs. PGC-1α restored these changes, whereas *Ppargc1a* knock-down further decreased *Tnfaip3* level. Interestingly, we found that both knock-down of *Ppargc1a* and *Drp1* in TGF-β1-treated RTECs did not reduce NLRP3 inflammasome activity, while RTECs with *Ppargc1a* overexpression and *Drp1* knock-down exhibited less activation of NLRP3 inflammasome. These findings suggest that PGC-1α can modulate NLRP3 signaling via other pathways such as TNFAIP3 beyond mitochondrial dynamics.

We used metformin as an indirect activator for PGC-1α. A pharmacologic dose of metformin can activate AMPK and increase ATP synthesis in various cells including renal intrinsic cells [52–55]. In this study, we used 1 mM of metformin based on previous studies showing that this dose of metformin decreased kidney fibrosis, ER stress, and activated AMPK activity. In the kidney of adenine-fed mice and RTECs treated with TGF-β1, the expression of p-AMPK was decreased. In contrast, metformin treatment reversed the decreased expression of p-AMPK. These changes were concordant to PGC-1α. With evidence that metformin activates the AMPK–PGC-1α axis, clinical studies on the beneficial effects of metformin against kidney injury are emerging. In a post-hoc analysis of the Trial to Reduce Cardiovascular Events with Aranesp Therapy (TREAT), metformin use was independently associated with a 23% lower risk of the kidney disease composite outcome [56]. In addition, a recent observational study by Kwon et al. showed that metformin use was associated with a significantly lower risk of end-stage kidney failure among 10,426 patients with type 2 diabetes [57]. The ability of metformin to activate AMPK has recently gained attention in polycystic kidney disease because this action of metformin decreased cell proliferation via inhibition of the mammalian target of rapamycin pathway and cyclic AMP levels in preclinical studies [58, 59]. Nevertheless, the use of metformin in advanced CKD carries a risk of lactic acidosis [60]. Thus, future trials should weigh the beneficial effects of metformin against lactic acidosis.

In conclusion, we demonstrated the role of PGC-1α in the regulation of the NLRP3 inflammasome activation via modulating mitochondrial dynamics and viability, and TNFAIP3 during kidney injury. These results suggest that inhibition of NLRP3 inflammasome by PGC-1α can be a future therapeutic target against CKD.

## Supplementary information

Supplementary Information

Fig. S1

Fig. S2

Fig. S3

Fig. S4

Fig S5

Fig. S6

Fig. S7

Fig. S8

Fig. S9

Fig. S10

Fig. S11

Full length WB Original Data File

aj-checklist

## Data Availability

The data supporting the findings of the present study are available from the corresponding author upon reasonable request.
