# Supplementary material for: PGC-1α inhibits the NLRP3 inflammasome via preserving mitochondrial viability to protect kidney fibrosis
Source: Cell Death Dis. 2022 Jan 10;13(1):31. doi: 10.1038/s41419-021-04480-3 (PMC8748677; doi:10.1038/s41419-021-04480-3)
Supplement: Supplementary file 1 — Supplementary Information [file 41419_2021_4480_MOESM1_ESM.docx]

**SUPPLEMENTARY INFORMATION**

**PGC-1α inhibits the NLRP3 inflammasome via preserving mitochondrial viability to protect kidney fibrosis**

Bo Young Nam Ph.D.^1,2*^, Jong Hyun Jhee, M.D., Ph.D.^3*^, Jimin Park M.S.^1,2^, Seonghun Kim Ph.D.^4,5^, Gyuri Kim^1,6^, Jung Tak Park, M.D., Ph.D.^1^, Tae-Hyun Yoo, M.D., Ph.D.^1^, Shin-Wook Kang, M.D., Ph.D.^1,6^, Je-Wook Yu M.D., Ph.D.^7^, and Seung Hyeok Han, M.D., Ph.D.^1^

^1^Department of Internal Medicine, College of Medicine, Institute of Kidney Disease Research, Yonsei University, Seoul, Korea

^2^Severance Biomedical Science Institute, College of Medicine, Yonsei University, Seoul, South Korea

^3^Division of Nephrology, Department of Internal Medicine, Gangnam Severance Hospital, Yonsei University College of Medicine, Seoul, Korea

^4^Oral Cancer Research Institute, Yonsei University College of Dentistry, Seoul, Korea.

^5^MET Life Science, Seoul, Korea.

^6^Department of Internal Medicine, College of Medicine, Severance Biomedical Science Institute, Brain Korea 21 PLUS, Yonsei University, Seoul, Korea

^7^Department of Microbiology and Immunology, Institute for Immunology and Immunological Diseases, Brain Korea 21 PLUS Project for Medical Science, Yonsei University College of Medicine, Seoul, Korea

**Corresponding Author:** Seung Hyeok Han, M.D., Ph.D.

Department of Internal Medicine, College of Medicine, Institute of Kidney Disease Research, Yonsei University, 50-1 Yonsei-ro, Seodaemun-gu, Seoul, Korea, 03722

Phone: 82-2-2228-1984/ Fax: 82-2-393-6884/ E-mail: hansh@yuhs.ac

**Table of contents**

**Supplementary Methods**

**Supplementary Table 1.** Sequences of oligonucleotide primers used for qPCR test

**Supplementary Figure legends**

**Supplementary Methods**

**Primary cell cultures**

The cells were cultured in the Dulbecco’s Modified Eagle Medium (DMEM) (Gibco, Thermo Fisher Scientific, Waltham, MA, USA) containing 10% fetal bovine serum (FBS) (Gibco, Thermo Fisher Scientific, Waltham, MA, USA), 100 U/ml penicillin G (Sigma-Aldrich, UK), 2.5 mg/ml amphotericin B (Sigma-Aldrich, UK), and 20 ng/ml epidermal growth factor (EGF) (Sigma-Aldrich, UK). In short, kidneys were dissected, placed in 1 ml ice-cold Dulbecco's Phosphate-Buffered Saline (DPBS) (Gibco, Thermo Fisher Scientific, Waltham, MA, USA), and minced into pieces of approximately 1 mm^3^. These pieces were transferred and digested for 60 minutes at 37°C, and the supernatants were sieved through a 100-mm nylon mesh. After centrifugation for 10 minutes at 3000 rpm, the pellet was resuspended in sterile red blood cell lysis buffer (8.26 g NH_4_Cl, 1 g KHCO_3_, and 0.037 g EDTA per 1 L double distilled H_2_O) and seeded in 10 cm culture dishes.

**Unilateral ureter obstruction mice model**

Unilateral ureter obstruction (UUO) was performed as described previously.^24^ In short, mice were anesthetized with Zoletil (10 mg/kg) (Virbac, Carros, France) and the left ureter was exposed via an incision under 0.5cm from costal margin. The mid-ureter was then obstructed using a ligation with silk sutures. The sham-operated mice underwent the same procedure without the obstruction of the ureter and used as controls. Both sham and UUO mice were daily treated with intraperitoneal injection of metformin (250 mg/kg). Mice were sacrificed at 5 days after UUO and the kidneys were removed while anesthetized.

**Total RNA extraction**

Whole kidney samples were rapidly frozen using liquid nitrogen and homogenized by mortar and pestle thrice with 700 μl of RNAiso reagent (Takara Bio Inc., Otsu, Shiga, Japan). For renal tubular epithelial cells (RTECs), 700 μl of RNAiso were added to the cell culture dish, and the suspension was collected and homogenized for 5 minutes at room temperature (RT). After then, 160 μl of chloroform was added into the homogenized samples of the kidneys and cells. Next, the mixture was shaken vigorously for 30 seconds, stored for 3 minutes at RT, and centrifuged 12,000 rpm for 15 minutes at 4°C. The aqueous phase located in the top of three phases was transferred to a fresh tube carefully not to be contaminated with the other phases. Extracted RNA was precipitated by adding 400 μl of isopropanol, and centrifuged at 12,000 rpm for 30 minutes at 4°C. The RNA pellet was washed with 70% ethanol, air-dried for 2 minutes, and dissolved in sterile diethyl pyrocarbonate (DEPC)-treated distilled water. The quantity and quality of extracted RNA were assessed by spectrophotometric measurements at wavelengths of 260 and 280 nm.

**Reverse transcription**

A Takara cDNA synthesis kit (Takara Bio Inc., Otsu, Shiga, Japan) was used to obtain first strand cDNA. Reverse transcription was conducted using 2 μg of total RNA extracts with 10 μM random hexanucleotide primer, 1 mM dNTP, 8 mM MgCl_2_, 30 mM KCl, 50 mM Tris-HCl at pH 8.5, 0.2 mM dithiothreithol, 25 U RNase inhibitor, and 40 U PrimeScript reverse transcriptase. The mixture was incubated for 10 minutes at 30°C, and for 1 hour at 42°C, followed by incubation for 5 minutes at 99°C for the inactivation of the enzyme.

**Real–time quantitative polymerase chain reaction**

The RNAs used for amplification were 25 ng per reaction tube. Using the ABI PRISM 7700 Sequence Detection System (Applied Biosystems, Foster City, CA, USA), a total volume of 20 μl mixture in each well was used containing 10 μl of SYBR Green PCR Master Mix (Applied Biosystems, Foster City, CA, USA), 5 μl of cDNA, and 5 pmol sense and antisense primers. The primer concentrations were determined by preliminary experiments that analyzed the optimal concentrations of each primer. The quantitative polymerase chain reaction (qPCR) conditions were as follows: 35 cycles of denaturation for 30 minutes at 94.5°C, annealing for 30 seconds at 60°C, and extension for 1 minute at 72°C. Initial heating for 9 minutes at 95°C and final extension for 7 minutes at 72°C were performed for all PCR reactions. The primer sequences used in this study were described in **Supplemental Table 1**. Each sample was run in triplicate in separated tubes and a control without cDNA was also run in parallel with each assay. After real-time PCR, the temperature was increased from 60 to 95°C at a rate of 2°C/minute to construct a melting curve. The cDNA content of each specimen was determined using a comparative CT method with 2^-△△CT^. The results were given as the relative expression normalized to the expression of 18s ribosomal RNA and expressed in arbitrary units.

**Western blot analyses**

Protein expression levels of the NLRP3 inflammasome pathway, oxidative stress marker, and profibrotic markers were examined with Western blot analyses. Harvested cultured cells and the mouse kidneys were lysed in sodium dodecyl sulfate (SDS) sample buffer [2% SDS, 10 mM Tris-HCl, pH 6.8, 10% (vol/vol) glycerol]. Lysate was centrifuged at 10,000 g for 10 minutes at 4°C, and the supernatant was stored at -70°C. Protein concentrations were determined with a Bio-Rad kit (Bio-Rad Laboratories, Inc., Hercules, CA, USA). Laemmli sample buffer was added to aliquots of 50 μg of the protein extracts, which were heated for 5 minutes at 100°C and electrophoresed in acrylamide denaturating SDS-polyacrylamide gel. A Hybond-ECL membrane was used to transfer protein using a Hoeffer semidry blotting apparatus (Hoeffer Instruments, San Francisco, CA, USA). After the protein was transferred to the membrane, it was incubated in blocking buffer containing PBS, 0.1% Tween-20, and 5% non-fat milk for 1 hour at RT, and then incubated overnight at 4°C in a 1:1,000 dilution of following polyclonal antibodies; AMPK (Cell Signaling Technology, MA, USA), PGC-1α (Abcam, Cambridge, MA, USA), Drp1 (BD Biosciences, San Jose, CA, USA), Mfn 1/2 (Cell Signaling Technology, MA, USA), NLRP3 (Adipogen Life Sciences, CA, USA), ASC (Cell Signaling Technology, MA, USA), IL-1β (Abcam, Cambridge, MA, USA), IL-18 (Abcam, Cambridge, MA, USA), tumor necrosis factor α induced protein 3, (TNFAIP3) (Cell Signaling Technology, MA, USA), fibronectin (DAKO, Carpentaria, CA, USA), type I collagen (Southern Biotech, Birmingham, AL, USA), Bax (Santa Cruz Biotechnology, Santa Cruz, CA, USA), B cell lymphoma–2 (Bcl–2) (Santa Cruz Biotechnology, Santa Cruz, CA, USA), Caspase-3 (Cell Signaling Technology, MA, USA), and β–actin (Sigma–Aldrich, UK). A horseradish peroxidase–conjugated anti–rabbit (Santa Cruz Biotechnology, Santa Cruz, CA, USA) or anti–mouse IgG antibody (Santa Cruz Biotechnology, Santa Cruz, CA, USA) were used as secondary antibodies. After frequent rinses, the membranes were developed by chemiluminescence (Gibco, Thermo Fisher Scientific, Waltham, MA, USA). To quantify the band densities, Image J software (National Institutes of Health, Bethesda, MD; online at http://rsbweb.nih.gov/ij) was used. The changes in the optical densities of bands from the treated groups relative to control cells or tissues were used for analysis.

**Histological and immunofluorescent staining**

To evaluate histologic features, formalin-fixed, paraffin-embedded kidney sections were stained with Masson’s trichrome reagent. All slide pictures were captured using an Olympus DP73 microscope.

**Enzyme-linked immunosorbent assay**

Levels of IL-1β and IL-18 in RTECs and the kidney tissues were determined using commercial enzyme-linked immunosorbent assay kits (R&D Systems, Minneapolis, MN, USA).

**Statistical analyses**

Statistical analyses were performed using IBM SPSS software for Windows version 23.0 (IBM Corporation, Armonk, NY, USA). Continuous variables are presented as mean ± standard deviation, and categorical variables are shown as numbers (percentage). To analyze differences between two groups, Mann–Whitney U test was used, and Kruskal–Wallis test was applied for comparison between more than two groups. For all analyses, *P* < 0.05 was considered statistically significant.

**Supplementary Table 1.** Sequences of oligonucleotide primers used for qPCR test

| **Genes** |  | **Sequences** |
| --- | --- | --- |
| *Ppargc1α* | **Forward** | AGTCCCATACACAACCGCAG |
|  | **Reverse** | CCCTTGGGGTCATTTGGTGA |
| *Mfn* | **Forward** | TTGGAAAACAGTGGGCTGGA |
|  | **Reverse** | AACGCTCTCTCTTTCGCACG |
| *Drp1* | **Forward** | GCTGCCTCAGATCGTCGTAG |
|  | **Reverse** | GGTGACCACACCAGTTCCTC |
| *Tfam* | **Forward** | GGAATGTGGAGCGTGCTAAAA |
|  | **Reverse** | TGCTGGAAAAACACTTCGGAATA |
| *mtDNA* | **Forward** | TTTTATCTGCATCTGAGTTT |
|  | **Reverse** | CCACTTCATCTTACCATTTA |
| *16s* | **Forward** | AGTAAGAACAAGCAAAGAT |
|  | **Reverse** | TCGTTTGGTTTCGGGGTTTC |
| *Nlrp3* | **Forward** | CTCCGGTTGGTGCTTAGACT |
|  | **Reverse** | TCCCAGACACTCATGTTGGC |
| *ASC* | **Forward** | GCACAGGCAAGCACTCATTG |
|  | **Reverse** | ACGAACTGCCTGGTACTGTC |
| *IL-1β* | **Forward** | ATCTCGCAGCAGCACATCAA |
|  | **Reverse** | AAGGTCCACGGGAAAGACAC |
| *IL-18* | **Forward** | CGGCCAAAGTTGTCTGATTCC |
|  | **Reverse** | ACTCTTGCGTCAACTTCAAGG |
| *FN* | **Forward** | TGACAACTGCCGTAGACCTGG |
|  | **Reverse** | TACTGGTTGTAGGTGTGGCCG |
| *Col1* | **Forward** | GAGCGGAGAGTACTGGATCG |
|  | **Reverse** | GCTTCTTTTCCTTGGGGTTC |
| *Bcl*–*2* | **Forward** | AGGAGCAGGTGCCTACAAGA |
|  | **Reverse** | GCATTTTCCCACCACTGTCT |
| *Bax* | **Forward** | TCCACCAAGAAGCTGAGCGAG |
|  | **Reverse** | GTCCAGCCCATGATGGTTCT |
| *18s* | **Forward** | CGCTTCCTTACCTGGTTGAT |
|  | **Reverse** | GGCCGTGCGTACTTAGACAT |

**Supplementary Figure legends**

**Figure S1. Bar graphs showing the changes in PGC-1α and NLRP3 inflammasome pathway in TGF-β1-treated RTECs and in adenine-fed mice (The results of Western blot analysis in Figure 1).** Protein expression levels of (A) PGC-1α were decreased, (B) NLRP3 inflammasome pathway were increased, and (C) fibrotic markers including fibronectin and collagen 1, and apoptotic cell death markers of Bax/bcl-2 and cleaved caspase-3 were increased in TGF-β1-treated RTECs. Protein expression levels of (D) PGC-1α were decreased, (E) NLRP3 inflammasome pathway were increased, and (F) fibrotic markers including fibronectin and collagen 1, and apoptotic cell death markers of Bax/bcl-2 and cleaved caspase-3 were increased in adenine-fed mice.

**Note:** * *P*<0.05 vs. control

**Abbreviations:** PGC-1α, peroxisomal proliferator-γ coactivator-1α; NLRP3, NOD-like receptor family, pyrin domain-containing 3; ASC, apoptosis-associated speck-like protein containing a caspase recruitment domain; RTEC, renal tubular epithelial cell; Ade, adenine.

**Figure S2. Bar graphs showing the changes in p-AMPK, PGC-1α and mitochondrial dynamics by restoration of PGC-1α.** (A, B) Protein expression levels of PGC-1α and mitochondrial dynamics were restored by *Ppargc1a* plasmid, whereas decreased by siPGC-1α in TGF-β1-treated RTECs. (C-E) Protein expression levels of p-AMPK, PGC-1α, and mitochondrial dynamics were restored by metformin in TGF-β1-treated RTECs. (F, G) The decreased mRNA and protein expression levels of PGC-1α by siPGC-1α was not recovered by metformin in TGF-β1-treated RTECs. The expression of p-AMPK was decreased in TGF-β-treated cells, whereas metformin treatment recovered this expression. (H, I) Disrupted mRNA and protein expression levels of mitochondrial dynamic-related genes were not altered by metformin in TGF-β1-treated RTECs with siPGC-1α. (J) Western blot images showing the changes of p-AMPK, PGC-1α, and mitochondrial dynamics in TGF-β1-treated RTECs by metformin after silencing *Ppargc1a.* (K-M) Protein expression levels of p-AMPK, PGC-1α, and mitochondrial dynamics were restored by metformin in RTECs from adenine-fed mice.

**Note:** * *P*<0.05 vs. control; #, ** *P*<0.05 vs. TGF-β or Ade.

**Abbreviations:** p-AMPK, phospho-AMP-activated kinase; PGC-1α, peroxisomal proliferator-γ coactivator-1α; RTEC, renal tubular epithelial cell; Ade, adenine.

**Figure S3. Alteration of mitochondrial functions with or without PGC-1α in TGF-β1-treated RTECs.** (A) Mitochondrial membrane potential was assessed with TMRE fluorescence in TGF-β1-treated RTECs. (B) Bar graph showing the intensity of TMRE. The intensity of TMRE was reduced by TGF-β1 treatment to RTECs, which was reversed by *Ppargc1a* plasmid transfection or metformin. (C) Mitochondrial respiration was evaluated by Seahorse analyzer in TGF-β1-treated RTECs. (D) Bar graphs showing the OCR assessed by Seahorse analyzed. OCRs were measured to analyze basal, spare respiratory capacity, proton leak respiration, and ATP production. The reduced mitochondrial respiration rate by TGF-β1 treatment in RTECs were reversed by *Ppargc1a* plasmid transfection or metformin.

**Note:** * *P*<0.05 vs. control; ** *P*<0.05 vs. TGF-β

**Abbreviations:** PGC-1α, peroxisomal proliferator-γ coactivator-1α; RTEC, renal tubular epithelial cell; TMRE, tetramethylrhodamine; OCR, oxygen consumption rate; met, metformin.

**Figure S4. PGC-1α attenuates mitochondrial damage in UUO animal model.** (A) mRNA and (B) protein expression levels of PGC-1α were increased in UUO mice with metformin. (C-E) mRNA expression levels of mitochondrial dynamic-related genes in UUO mice were restored with metformin. (F) Transmission electron microscopy images of RTECs from UUO mice showed restoration of mitochondrial structure with metformin.

**Note:** * *P*<0.05 vs. control; #, ** *P*<0.05 vs. UUO.

**Abbreviations:** PGC-1α, peroxisomal proliferator-γ coactivator-1α; UUO, unilateral ureteral obstruction; Met, metformin; Mfn, mitofusin; Drp1, dynamin related protein 1; Tfam, mitochondrial transcriptional factor A.

**Figure S5. Bar graphs showing the changes in fibrotic markers by restoration of PGC-1α (The results of Western blot analysis in Figure 4).** (A-C) Protein expression levels of fibrotic markers including fibronectin and collagen 1, and apoptotic cell death markers of Bax/bcl-2 and cleaved caspase-3 were reduced by *Ppargc1a* plasmid or metformin, which was reversed by siPGC-1α in TGF-β1-treated RTECs and adenine-fed mice.

**Note:** * *P*<0.05 vs. control; #, ** *P*<0.05 vs. TGF-β or Ade.

**Abbreviations:** PGC-1α, peroxisomal proliferator-γ coactivator-1α; RTEC, renal tubular epithelial cell; Ade, adenine.

**Figure S6. PGC-1α protects kidney fibrosis and attenuates activation of NLRP3 inflammasome in UUO animal model.** (A) mRNA and (B) protein expression levels of fibrotic and apoptotic markers in UUO mice were reduced with metformin. (C) mRNA and (D) protein expression levels of NLRP3 inflammasome pathway in UUO mice were reduced with metformin. (E) Decreased oxidative stress levels by MDA were observed in UUO mice with metformin. (F) mRNA expression level of *tnfaip3* in UUO was increased with metformin.

Note: * *P*<0.05 vs. control; ** *P*<0.05 vs. UUO.

**Abbreviations:** PGC-1α, peroxisomal proliferator-γ coactivator-1α; NLRP3, NOD-like receptor family, pyrin domain-containing 3; ASC, apoptosis-associated speck-like protein containing a caspase recruitment domain; UUO, unilateral ureteral obstruction; Met, metformin; MDA, malondialdehyde; TNFAIP3, tumor necrosis factor α induced protein 3.

**Figure S7. Changes in mitochondrial dynamics, NLRP3 inflammasome pathway, and fibrotic markers with a pan-caspase inhibitor in TGF-β1-treated RTECs.** (A) The reduced mRNA level of *Ppargc1a* were reversed by pan-caspase inhibitor in RTECs. (B)The altered mRNA expression levels of mitochondrial dynamic-related genes in TGF-β1-treated RTECs were restored with pan-caspase inhibitor. (C) The increased mRNA levels of *nlrp3* inflammasome pathway were attenuated by pan-caspase inhibitor in RTECs. (D) The reduced mRNA levels of fibrotic and apoptotic cell injury markers were reversed by pan-caspase inhibitor in RTECs.

**Note:** * *P*<0.05 vs. control; ** *P*<0.05 vs. TGF-β1.

**Abbreviations:** PGC-1α, peroxisomal proliferator-γ coactivator-1α; Met, metformin; Mfn, mitofusin; Drp1, dynamin related protein 1; Tfam, mitochondrial transcriptional factor A; NLRP3, NOD-like receptor family, pyrin domain-containing 3; ASC, apoptosis-associated speck-like protein containing a caspase recruitment domain.

**Figure S8. Bar graphs showing the changes in NLRP3 inflammasome pathway and TNFAIP3 by restoration of PGC-1α (The results of Western blot analysis in Figure 5 and 8).** (A and B) Protein expression levels of NLRP3 inflammasome pathway were restored by *Ppargc1a* plasmid and metformin whereas decreased by siPGC-1α in TGF-β1-treated RTECs (C) Protein expression levels of NLRP3 inflammasome pathway were restored by metformin in adenine-fed mice. (D and E) Protein expression levels of TNFAIP3 were restored by *Ppargc1a* plasmid and metformin whereas decreased by siPGC-1α in TGF-β1-treated RTECs (F) Protein expression levels of TNFAIP3 were restored by metformin in adenine-fed mice.

**Note:** * *P*<0.05 vs. control; # *P*<0.05 vs. TGF-β or Ade.

**Abbreviations:** PGC-1α, peroxisomal proliferator-γ coactivator-1α; NLRP3, NOD-like receptor family, pyrin domain-containing 3; ASC, apoptosis-associated speck-like protein containing a caspase recruitment domain; RTEC, renal tubular epithelial cell; Ade, adenine.

**Figure S9. Inhibition of *Drp1* and activation of NLRP3 inflammasome pathway with or without PGC-1α.** (A) *Drp1* was knockdowned with lentivirus containing *Drp1* targeting short hairpin RNA (shRNA) in TGF-β1-treated RTECs. (B) The inhibition of *Drp1* induced activation of NLRP3 inflammasome pathway in TGF-β1-treated RTECs, which were restored by *Ppargc1a* plasmid transfection.

**Note:** * *P*<0.05 vs. control; ** *P*<0.05 vs. TGF-β1; *** *P*<0.05 vs. TGF-β1 with LvDrp1.

**Abbreviations:** Drp1, dynamin related protein 1; shRNA, short hairpin RNA; PGC-1α, peroxisomal proliferator-γ coactivator-1α; Tfam, mitochondrial transcriptional factor A; NLRP3, NOD-like receptor family, pyrin domain-containing 3; ASC, apoptosis-associated speck-like protein containing a caspase recruitment domain; LvDrp1, lentivirus containing Drp1 targeting shRNA.

**Figure S10. The absence of *Nlrp3* improves mitochondrial dynamics and cell death.** (A, B) mRNA and (C) protein expression levels of PGC-1α and mitochondrial dynamics in *Nlrp3*^-/-^ mice with TGF-β1 treatment. (D, E) mRNA and protein expression levels of NLRP3 inflammasome pathway in *Nlrp3*^-/-^ mice with TGF-β1 treatment. (F, G) mRNA and protein expression levels of fibrotic and apoptotic markers in *Nlrp3*^-/-^ mice with TGF-β1 treatment **Note:** * *P*<0.05 vs. control; ** *P*<0.05 vs. TGF-β1; *** *P*<0.05 vs. *Nlrp3*^-/-^ mice.

**Abbreviations:** PGC-1α, peroxisomal proliferator-γ coactivator-1α; Mfn, mitofusin; Drp1, dynamin related protein 1; Tfam, mitochondrial transcriptional factor A; NLRP3, NOD-like receptor family, pyrin domain-containing 3; ASC, apoptosis-associated speck-like protein containing a caspase recruitment domain.

**Figure S11. Schematic summary.** PGC-1α regulates the NLRP3 inflammasome activation via modulating mitochondrial viability and dynamics, and TNFAIP3 during kidney cell death and fibrosis.

***Note:*** Black and red arrows indicate stimulus effect toward objects; black and red blunted arrows indicate inhibitory effect toward objects.

**Abbreviations:** PGC-1α, peroxisomal proliferator–γ coactivator-1α; NLRP3, NOD-like receptor family, pyrin domain-containing 3 TNFAIP3, tumor necrosis factor α induced protein 3; mROS, mitochondrial generated ROS.
