# Supplementary figures and images for: PGC-1α inhibits the NLRP3 inflammasome via preserving mitochondrial viability to protect kidney fibrosis
Source: Cell Death Dis. 2022 Jan 10;13(1):31. doi: 10.1038/s41419-021-04480-3 (PMC8748677; doi:10.1038/s41419-021-04480-3)

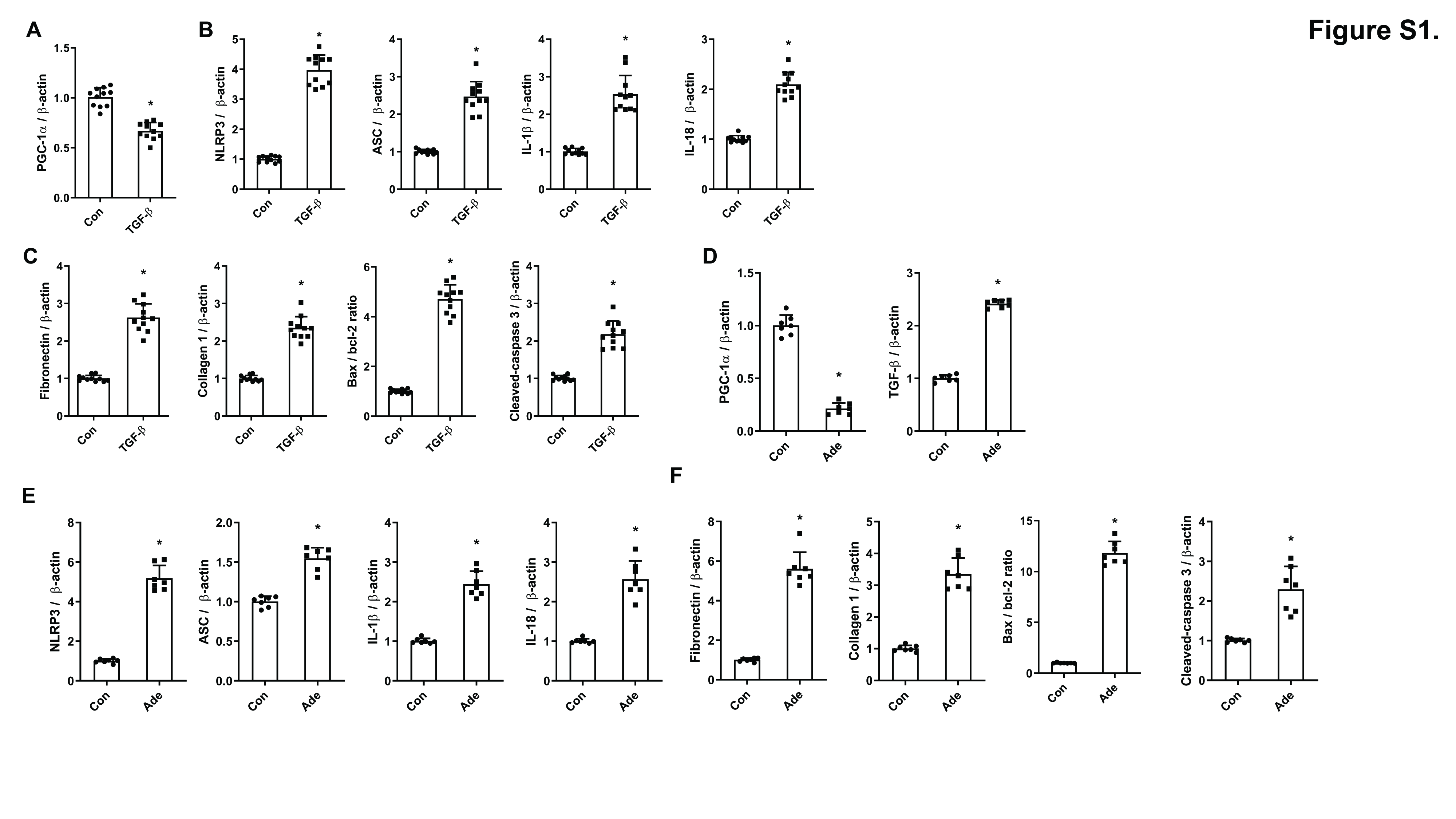

Supplement: Supplementary file 2 — Fig. S1 [file 41419_2021_4480_MOESM2_ESM.tif]

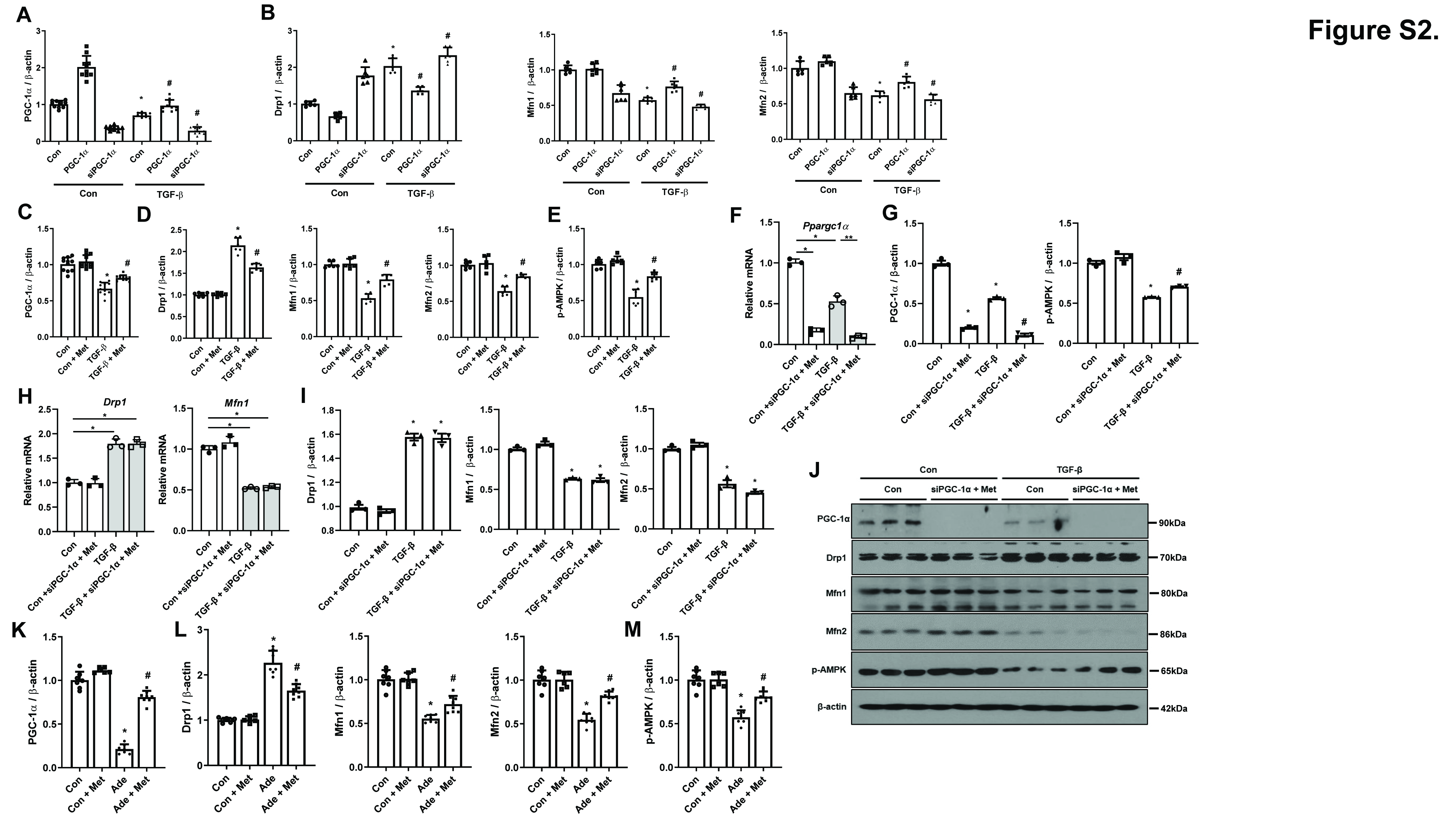

Supplement: Supplementary file 3 — Fig. S2 [file 41419_2021_4480_MOESM3_ESM.tif]

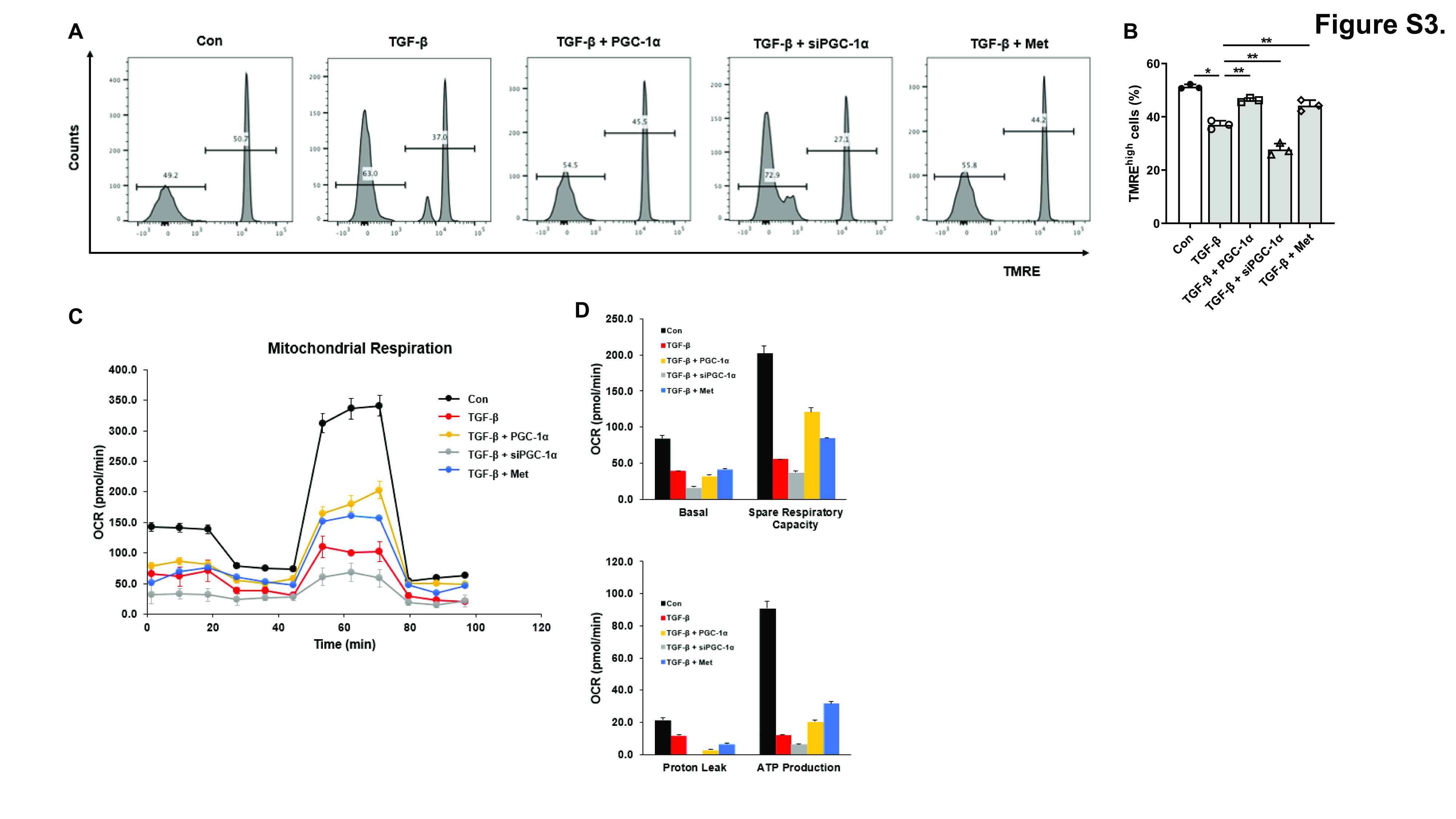

Supplement: Supplementary file 4 — Fig. S3 [file 41419_2021_4480_MOESM4_ESM.tif]

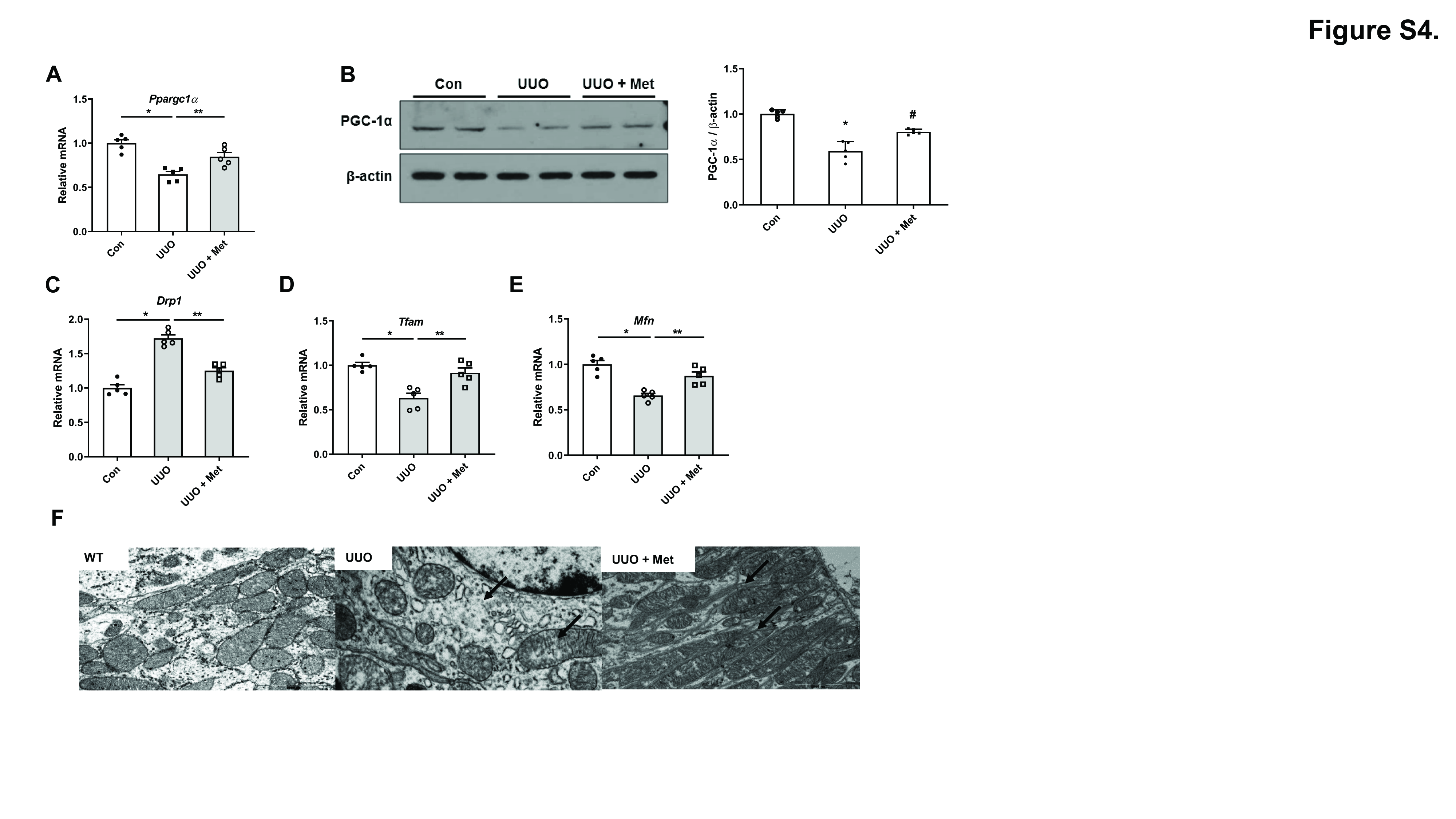

Supplement: Supplementary file 5 — Fig. S4 [file 41419_2021_4480_MOESM5_ESM.tif]

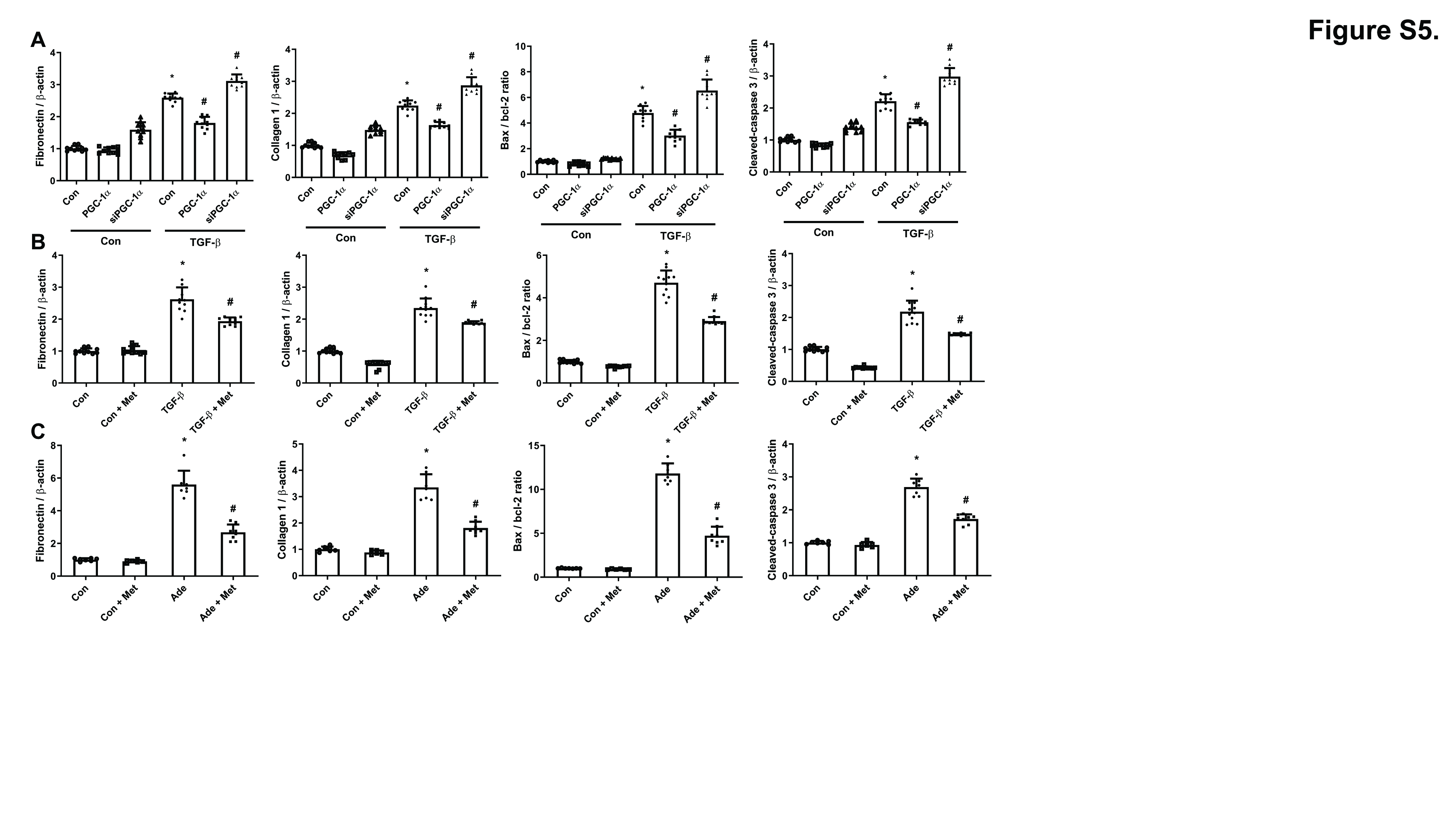

Supplement: Supplementary file 6 — Fig S5 [file 41419_2021_4480_MOESM6_ESM.tif]

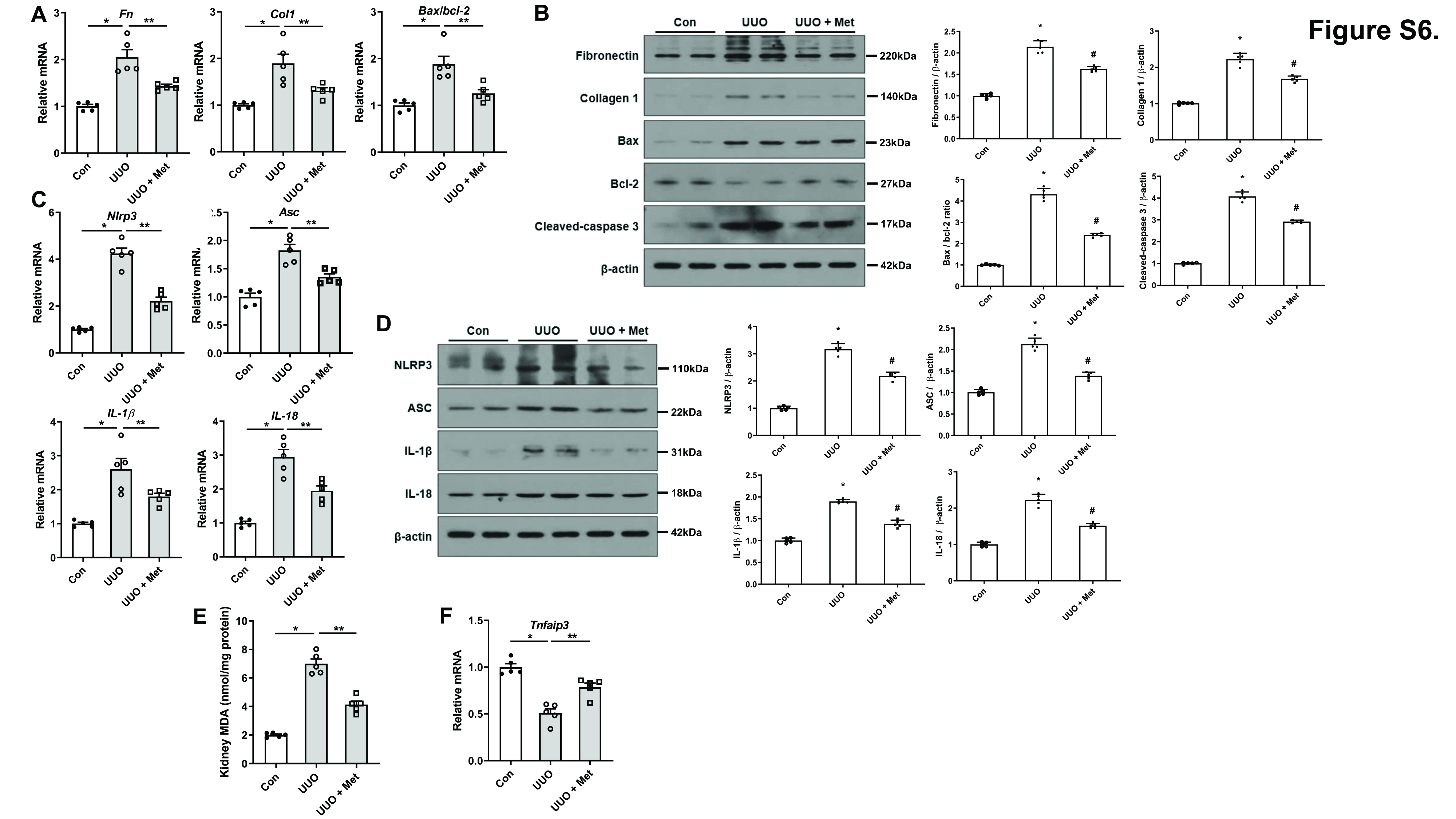

Supplement: Supplementary file 7 — Fig. S6 [file 41419_2021_4480_MOESM7_ESM.tif]

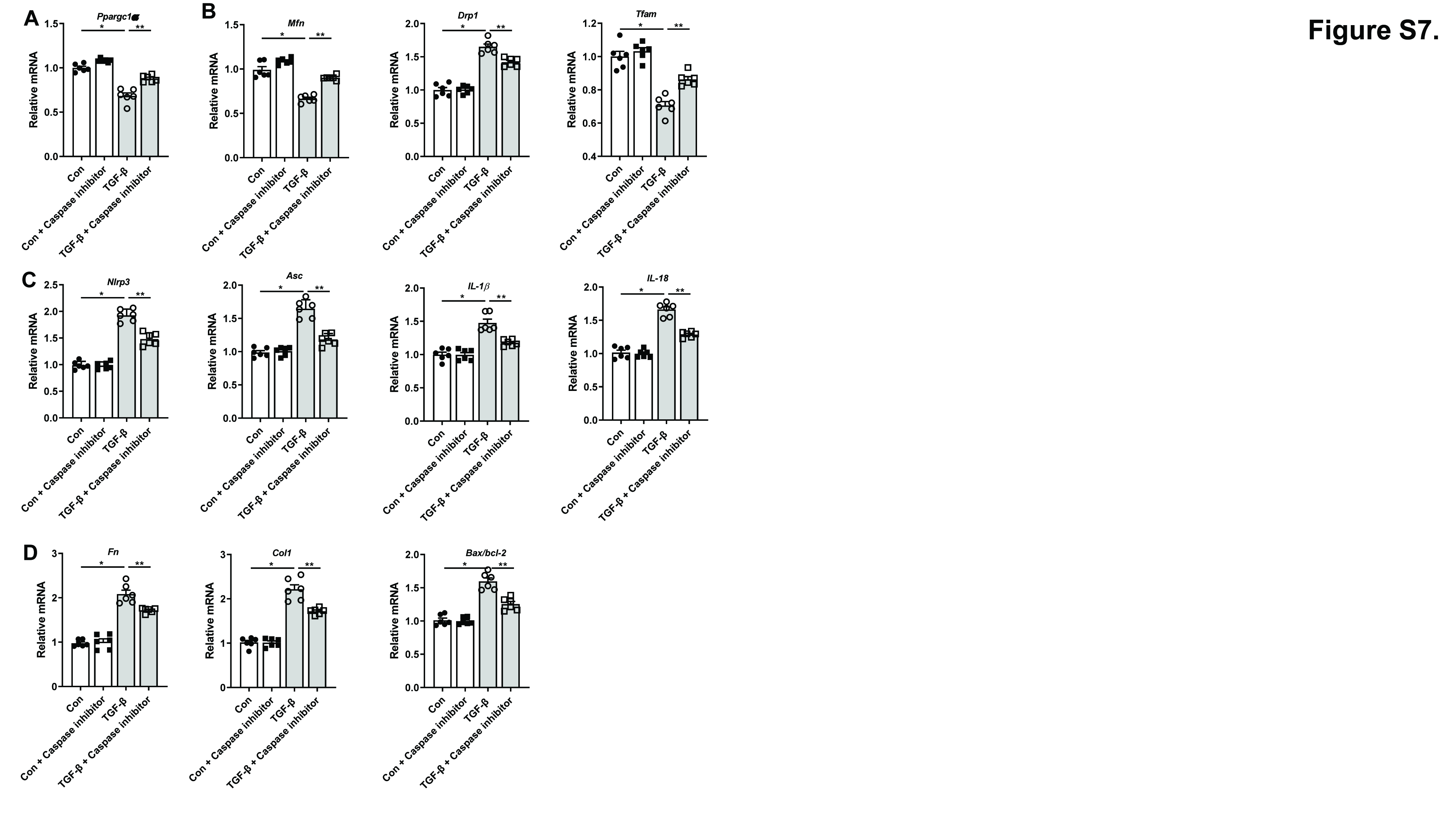

Supplement: Supplementary file 8 — Fig. S7 [file 41419_2021_4480_MOESM8_ESM.tif]

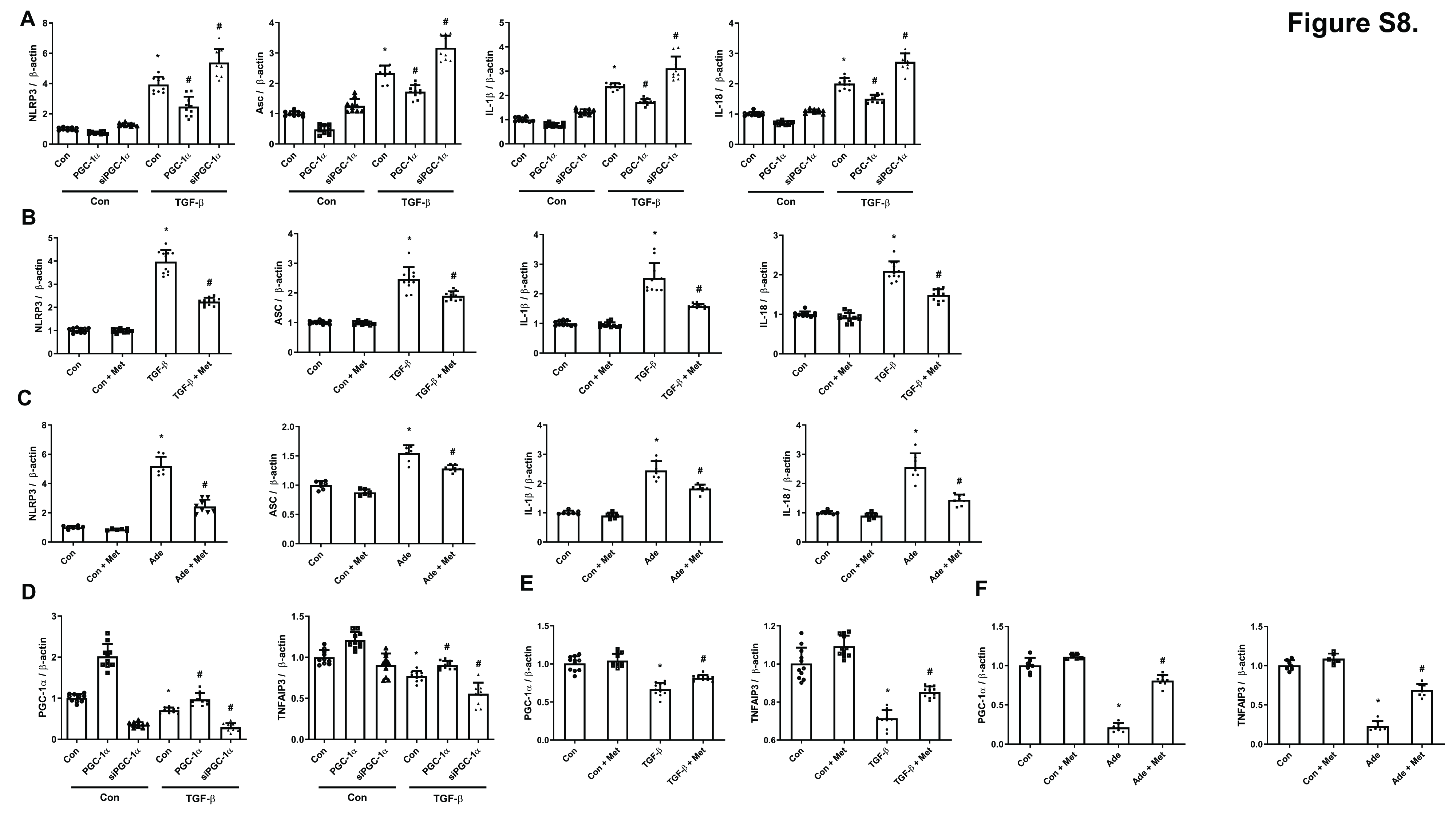

Supplement: Supplementary file 9 — Fig. S8 [file 41419_2021_4480_MOESM9_ESM.tif]

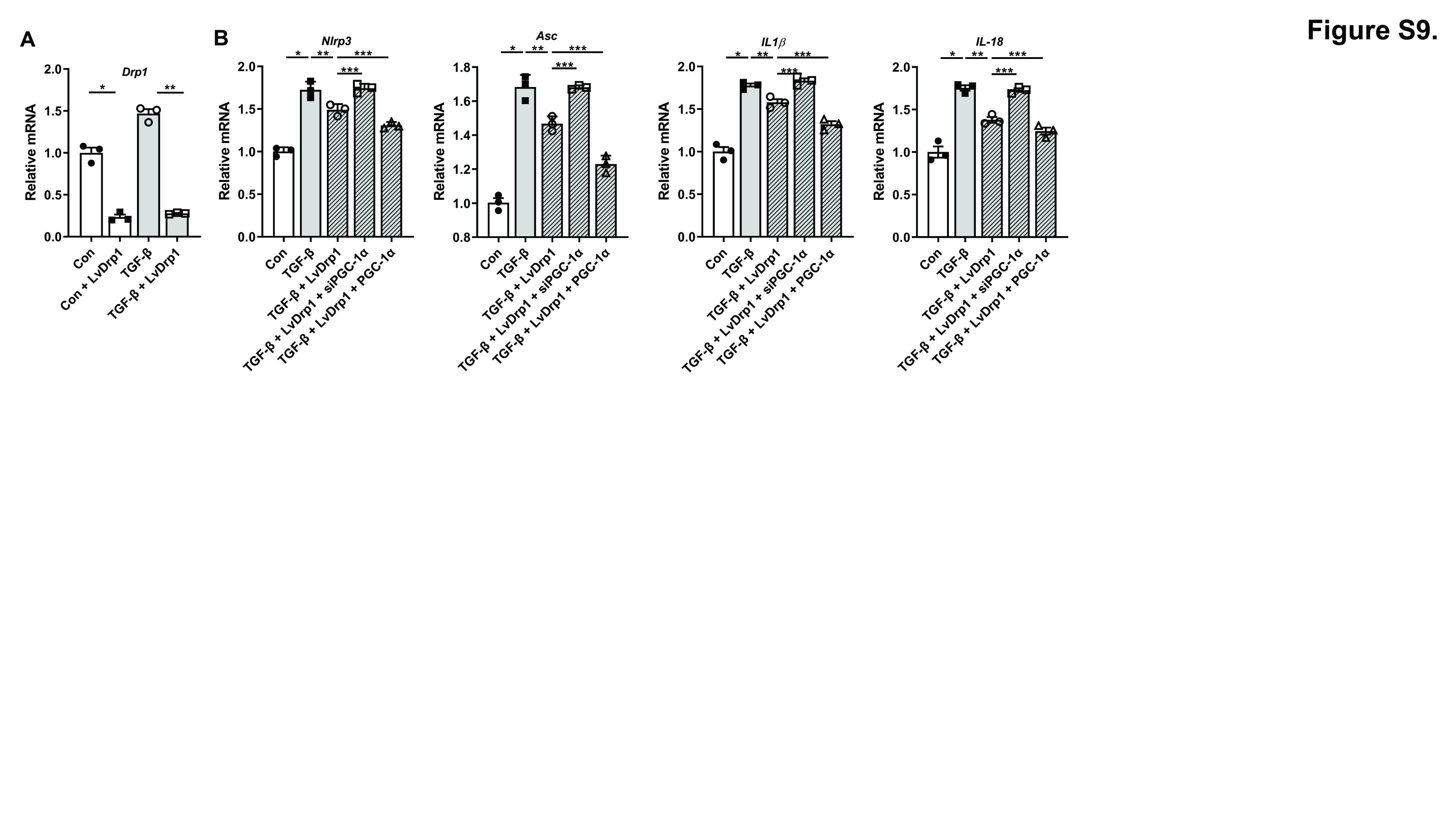

Supplement: Supplementary file 10 — Fig. S9 [file 41419_2021_4480_MOESM10_ESM.tif]

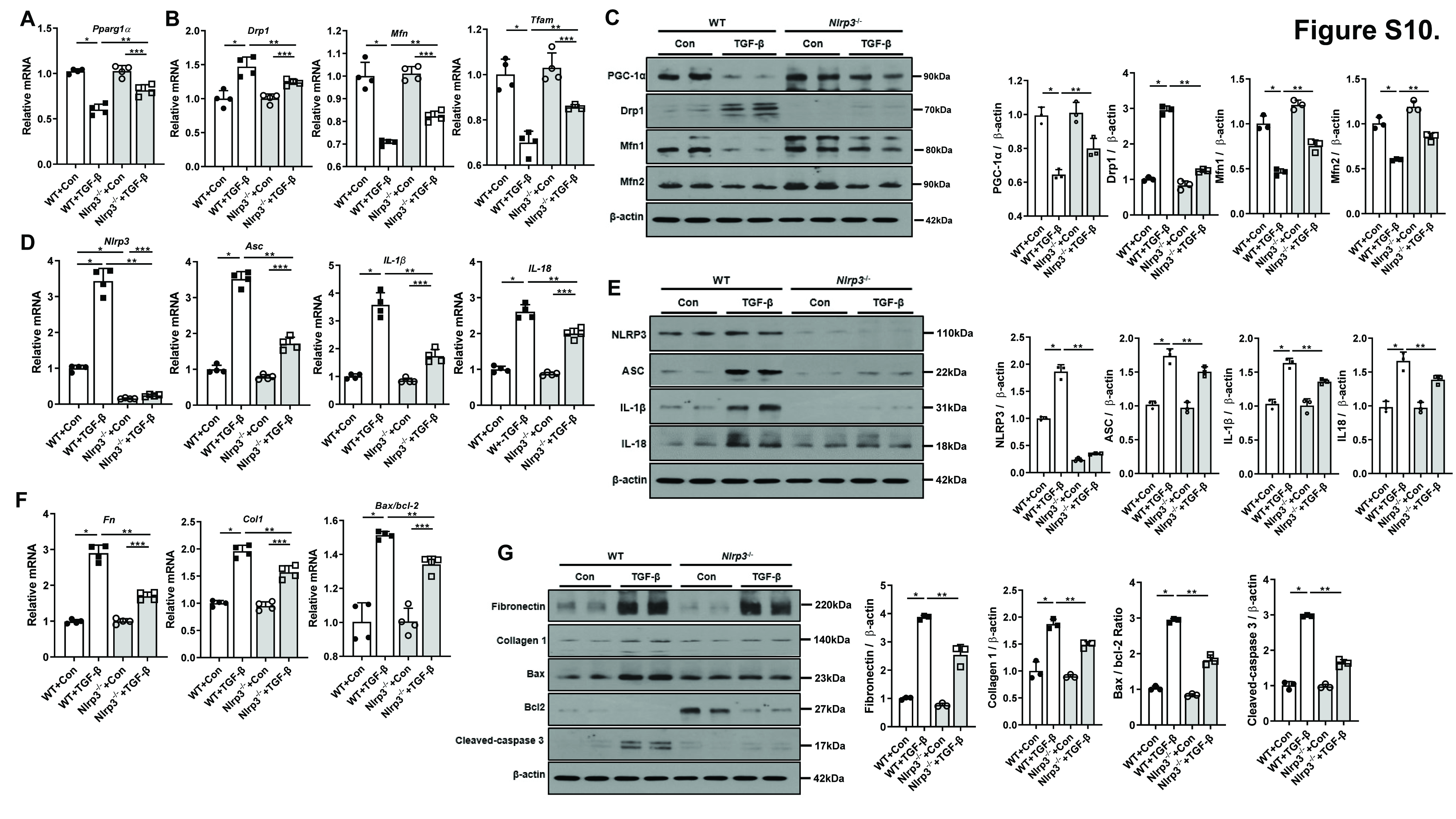

Supplement: Supplementary file 11 — Fig. S10 [file 41419_2021_4480_MOESM11_ESM.tif]

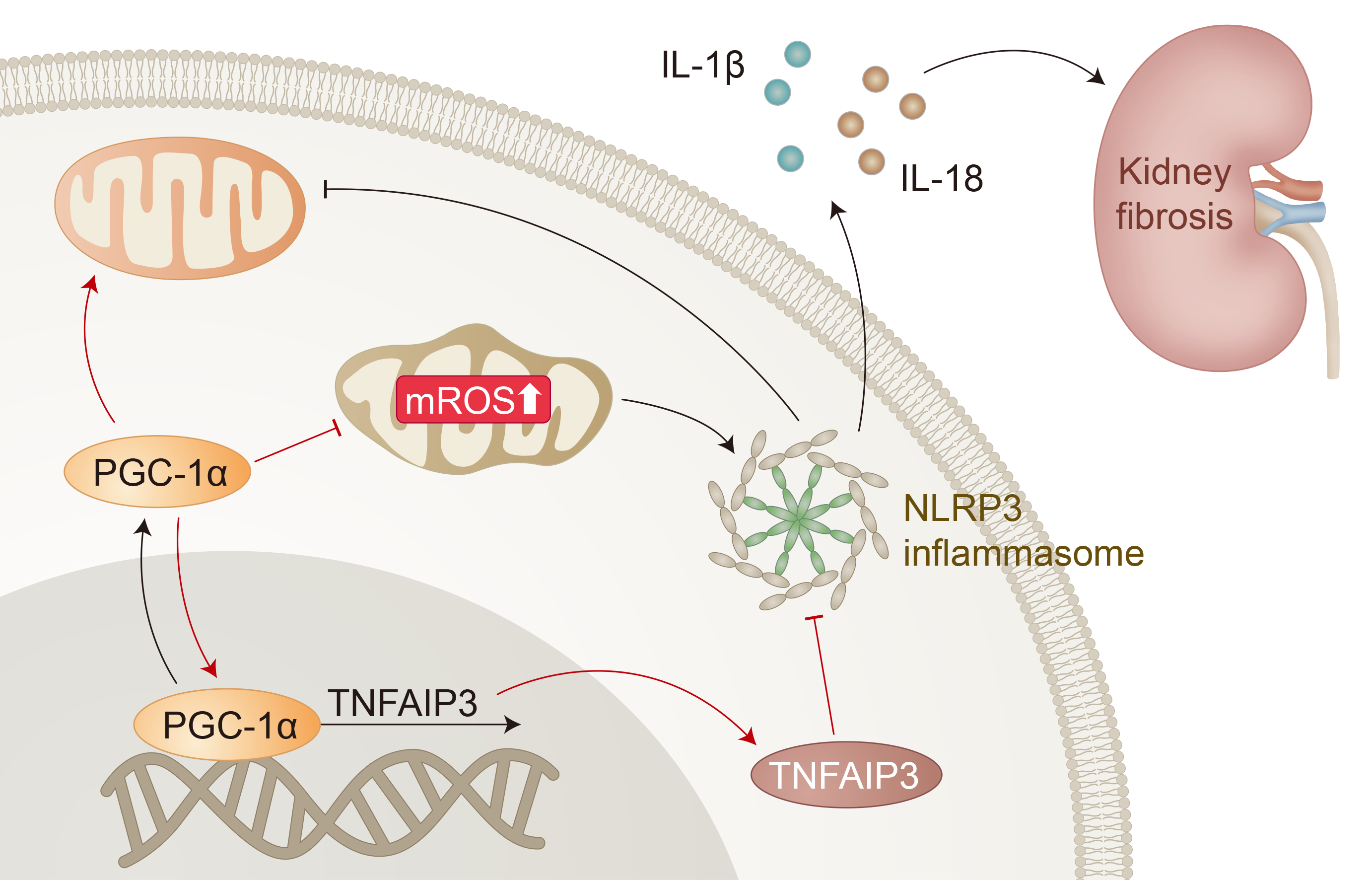

Supplement: Supplementary file 12 — Fig. S11 [file 41419_2021_4480_MOESM12_ESM.tif]
