# Supplementary material for: PGC-1α inhibits the NLRP3 inflammasome via preserving mitochondrial viability to protect kidney fibrosis
Source: Cell Death Dis. 2022 Jan 10;13(1):31. doi: 10.1038/s41419-021-04480-3 (PMC8748677; doi:10.1038/s41419-021-04480-3)
Supplement: Supplementary file 13 — Full length WB Original Data File [file 41419_2021_4480_MOESM13_ESM.pdf]

Full length WB (Figure 1)

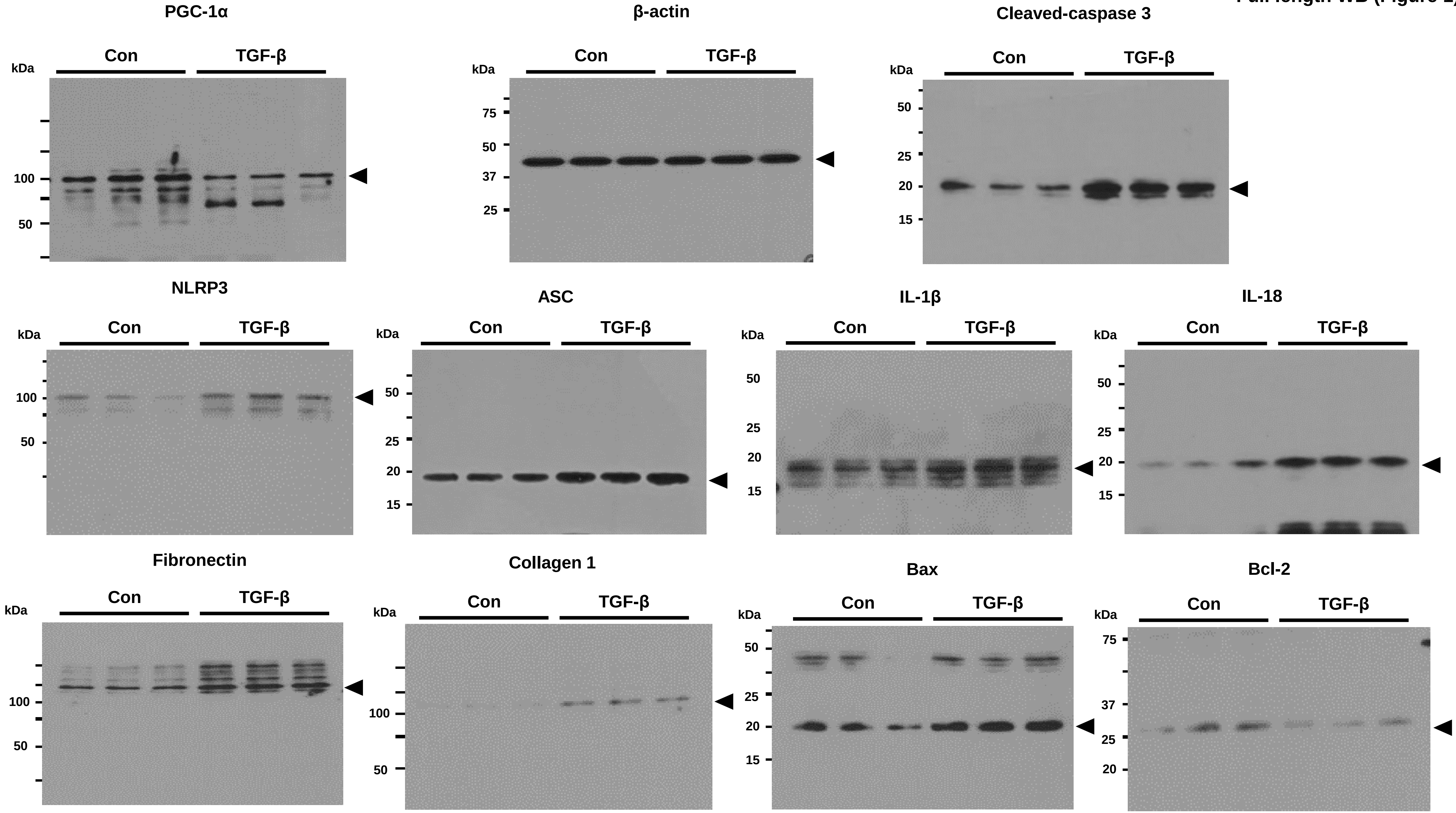

Full length WB (Figure 2)

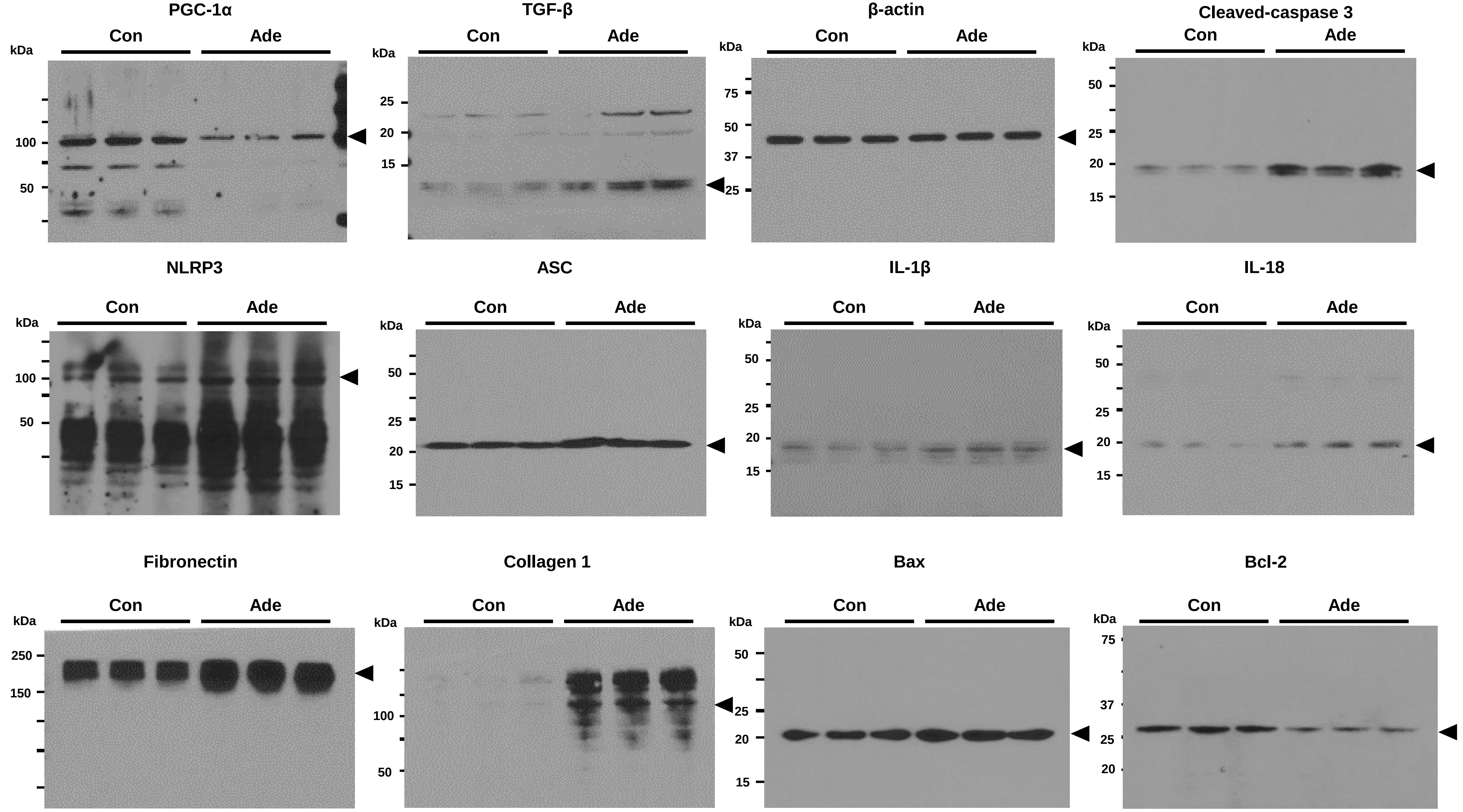

Full length WB (Figure 3)

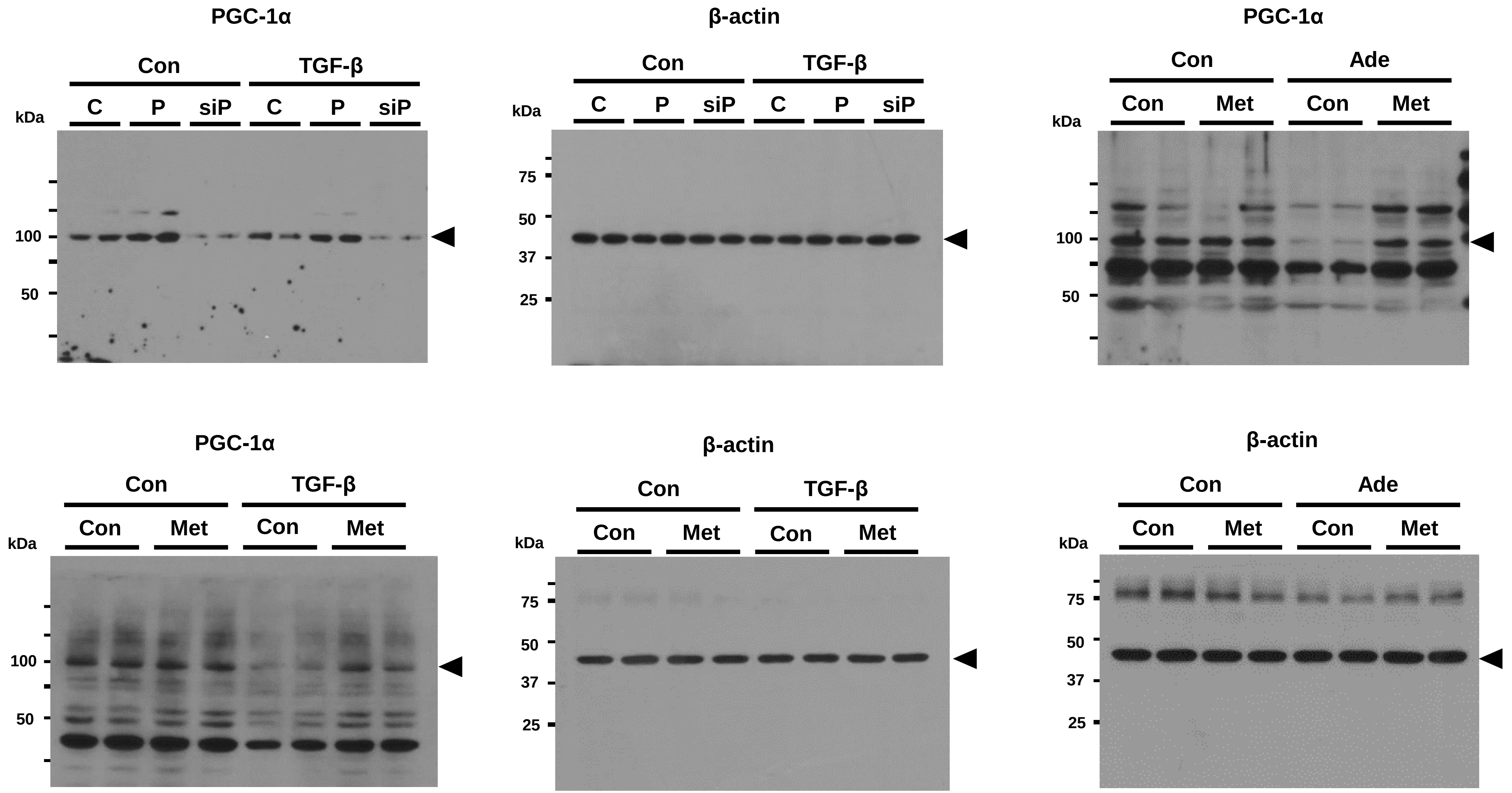

Abbreviation: C=control, P=PGC-1α, siP=siPGC-1α

Full length WB (Figure 4)

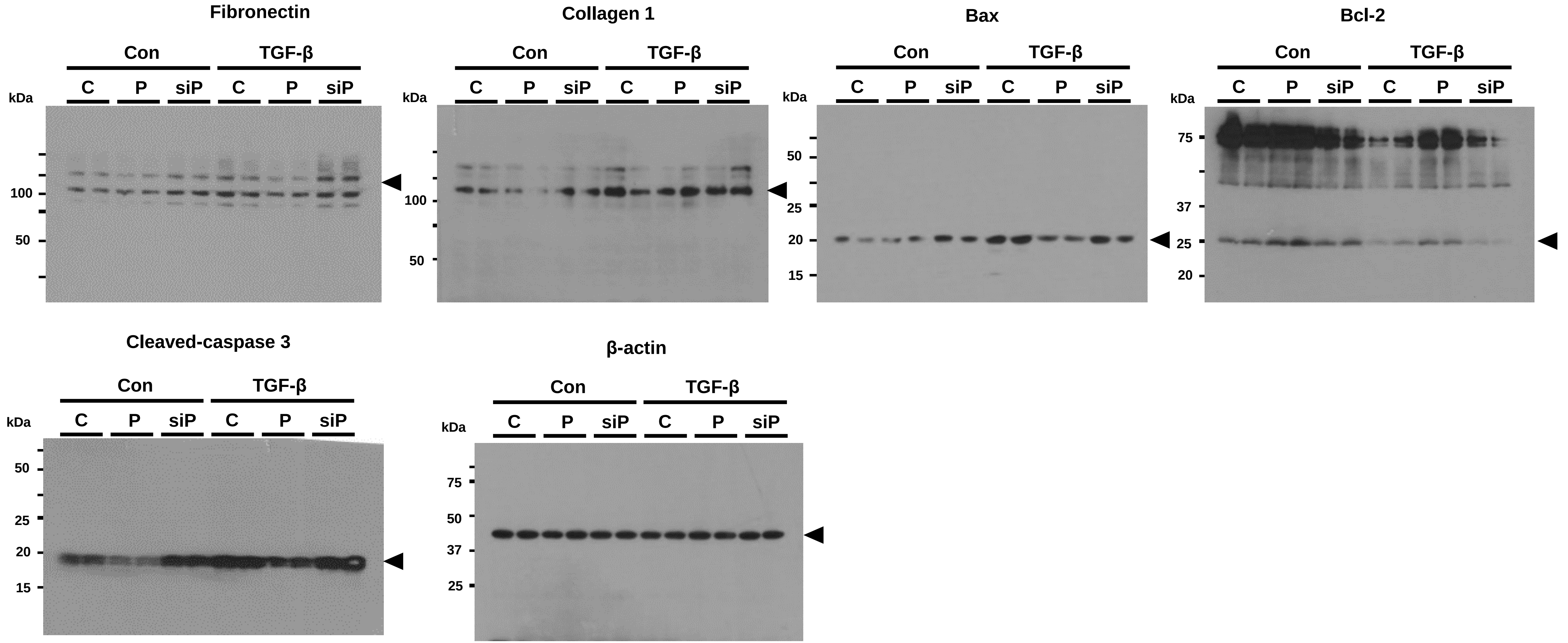

Abbreviation: C=control, P=PGC-1α, siP=siPGC-1α

Full length WB (Figure 4)

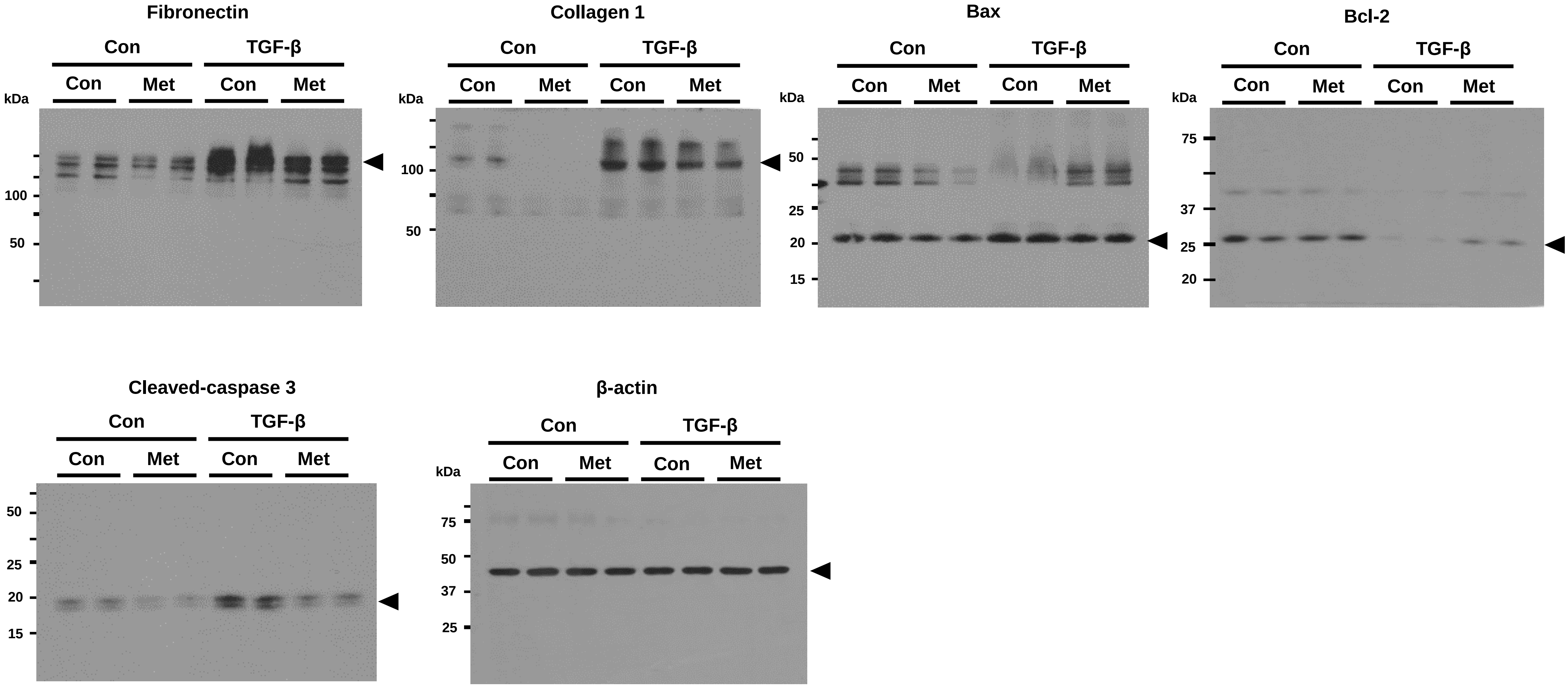

Full length WB (Figure 4)

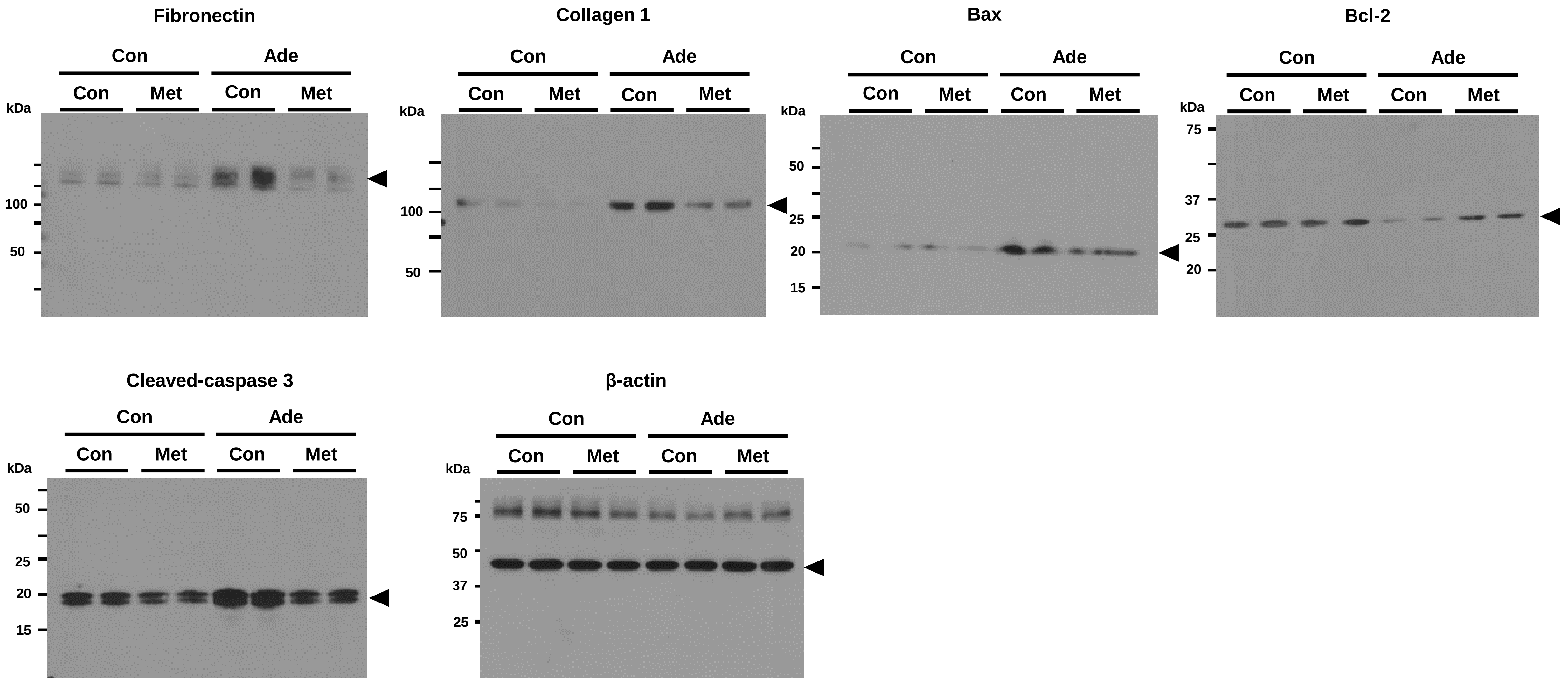

Full length WB (Figure 5)

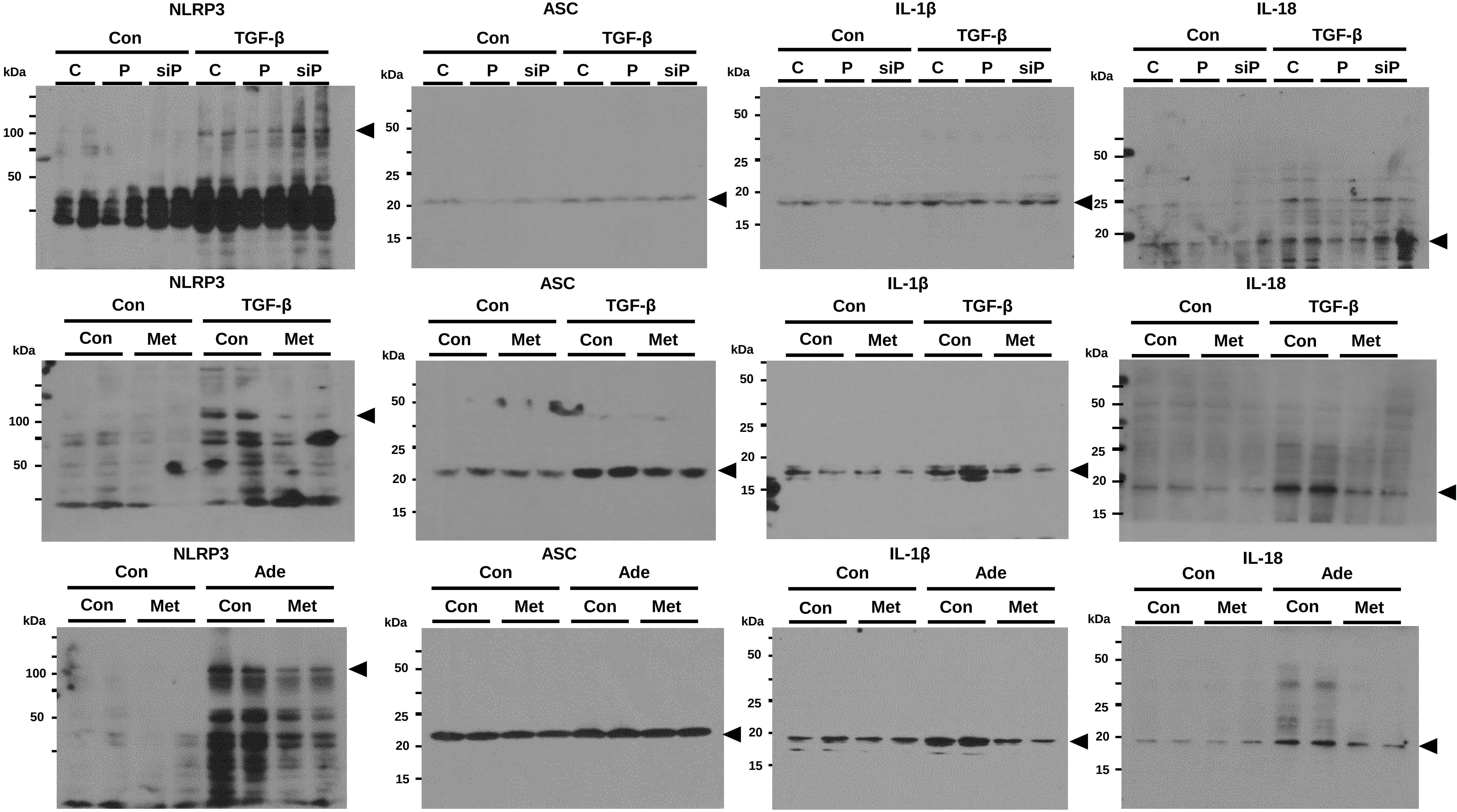

Full length WB (Figure 8)

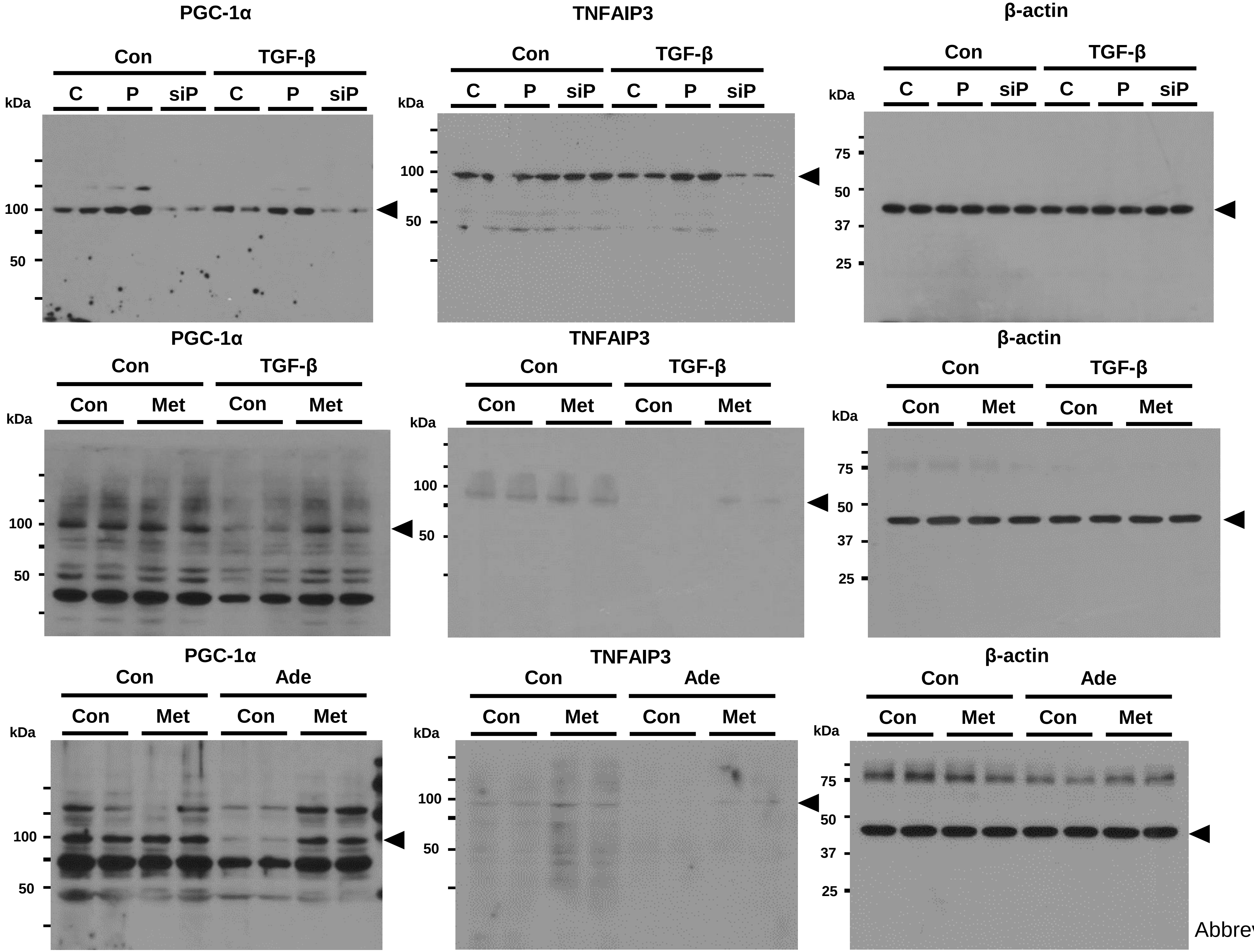

Abbreviation: C=control, P=PGC-1α, siP=siPGC-1α
